# Oral Food Challenges to Milk and Egg in Children: Associations with Skin Prick Tests and IgE Sensitization Profiles

**DOI:** 10.3390/nu18132157

**Published:** 2026-07-03

**Authors:** Joanna Zielińska, Karolina Dumycz, Maria Wawszczak, Agnieszka Szczukocka, Patrycja Krzosek-Ptak, Zofia Bojakowska, Marek Kulus, Katarzyna Grzela

**Affiliations:** 1 Department of Pediatric Pneumonology and Allergy, Medical University of Warsaw, 02-091 Warsaw, Poland; 2 Faculty of Medicine, Medical University of Warsaw, 02-091 Warsaw, Poland

**Keywords:** cow’s milk allergy, hen’s egg allergy, oral food challenge, skin prick test, specific IgE

## Abstract

Background: Oral food challenge (OFC) remains the gold standard for diagnosing food allergy; however, it is time-consuming and associated with the risk of allergic reactions. The identification of reliable biomarkers capable of predicting OFC outcomes could improve patient selection and reduce the number of OFCs required in clinical practice. Among the most widely used biomarkers are skin prick tests (SPTs) and serum-specific IgE (sIgE) measurements, including component-resolved diagnostics. However, their diagnostic utility, as well as the comparability of different diagnostic platforms, remains incompletely defined in cow’s milk (CM) and hen’s egg (HE) allergy. Methods: We retrospectively analyzed results of diagnostic tests obtained in children undergoing OFCs to baked milk, raw milk, baked egg, and boiled egg. Diagnostic performance of SPTs to either allergen extracts or native food allergens, as well as sIgE measured using ImmunoCAP and ALEX2 platforms, was evaluated using receiver operating characteristic (ROC) analysis and logistic regression models. Results: Diagnostic performance varied across allergy phenotypes. SPTs to native food allergens demonstrated good discriminative capacity for raw milk and boiled egg allergy, whereas SPTs to standardized extracts showed limited diagnostic utility overall. In baked milk allergy, casein-sIgE measured by ImmunoCAP demonstrated the best diagnostic performance. In raw milk allergy, α-lactalbumin-sIgE measured using ALEX2 platform and β-lactoglobulin-sIgE measured by ImmunoCAP showed greater diagnostic relevance than casein-sIgE. In boiled egg allergy, SPT to raw egg yolk demonstrated the highest diagnostic value. In baked egg allergy, ovalbumin-sIgE measured using the ALEX2 platform showed the best performance, while ovomucoid-sIgE did not demonstrate clear superiority. ALEX2 showed good concordance with ImmunoCAP despite systematic differences in absolute sIgE values. Combined multivariable models provided only modest improvements over single predictors and did not achieve sufficient accuracy to replace OFCs. Conclusions: SPTs to either allergen extracts or native food allergens, as well as sIgE measurements, demonstrate moderate and heterogeneous utility in predicting OFC outcomes for CM and HE allergy. Although ALEX2 and ImmunoCAP showed comparable overall performance, platform-specific interpretation may be required. OFC remains indispensable for definitive diagnosis.

## 1. Introduction

Cow’s milk (CM) and hen’s egg (HE) allergies are common conditions, with self-reported rates of 5.7% and 2.4%, respectively, whereas the prevalence confirmed by oral food challenge (OFC) is considerably lower, at 0.3% and 0.8%, respectively [1]. Similar prevalence rates have been reported in the Polish pediatric population, where both CM and HE allergy affect approximately 0.6% of children younger than two years of age [2,3]. The marked discrepancy between self-reported and OFC-confirmed prevalence highlights the limitations of diagnosis based solely on clinical history and underscores the importance of objective diagnostic evaluation. Both conditions predominantly affect the pediatric population, particularly youngest children [1,4]. Food allergy symptoms in early childhood often resolve spontaneously, with tolerance developing in up to 92% of children with CM allergy by 5 years of age and in up to 89% of children with HE allergy by 6 years of age [5,6,7]. These findings highlight the importance of regular clinical reevaluation to assess the development of tolerance [5].

Thermal processing and the food matrix effect associated with baking reduce the allergenicity of milk and egg proteins, which may result in tolerance to these forms in some allergic children [8]. Introduction of thermally processed milk or egg into the diet at an appropriate time may significantly improve both quality of life and nutritional status of affected patients [9]. Moreover, evidence suggests that regular ingestion of tolerated baked milk products may accelerate the development of tolerance to unheated milk in children with CM allergy [10]. Although a similar effect has been proposed for baked egg ingestion in children with raw egg allergy, currently evidence remains limited due to the small number of participants included in previous studies and further research is required [11].

The OFC remains the gold standard for confirming allergy or tolerance to a specific food [12]. Despite being the reference standard, OFC is not universally performed in all eligible patients in routine clinical practice due to its resource-intensive nature, requirement for hospital-based supervision, limited availability, and risk of allergic reactions requiring medical intervention [13]. Consequently, access to OFC may be delayed, and clinicians often rely on surrogate biomarkers for pre-referral risk stratification. Improved risk stratification may therefore support more appropriate selection of patients for OFC, enhance safety, reduce unnecessary procedures, and optimize healthcare resources. In addition, earlier identification of patients eligible for OFC may facilitate timely introduction of tolerated baked or unheated milk and egg products, with potential benefits for diet quality and patient well-being.

Numerous diagnostic tests are widely available in clinical practice, including skin prick tests (SPTs), food-specific IgE (sIgE) measurements, and component-resolved diagnostics; however, their diagnostic performance is variable, interpretation remains challenging, and the comparative utility of different allergen components-specific IgE and diagnostic platforms is still insufficiently defined [5].

A recent meta-analysis [14] showed that SPTs provide high sensitivity for the diagnosis of CM and HE allergy, whereas sIgE measurements generally offer higher specificity. However, data on predicting OFC outcomes for baked milk and baked egg allergy remain limited [14].

The basophil activation test (BAT) may be useful in predicting OFC outcomes, particularly in children under 2 years of age; however, it is not widely available and is more expensive than sIgE measurement and SPTs [15,16,17]. An alternative approach may involve combining different biomarkers, using more accessible and less expensive tests initially while reserving BAT for diagnostically uncertain cases [16].

Component-resolved diagnostics represent another promising strategy in allergy evaluation. The value of casein-sIgE may serve as a cost-effective predictor of OFC outcomes with baked milk and even of reaction severity; however, reported cut-off values vary substantially across studies and are influenced by patient age [17,18,19]. Furthermore, not all studies have confirmed the utility of casein-sIgE [20]. Similarly, the role of ovomucoid-sIgE in predicting OFC outcomes in baked egg allergy remains unclear; while some studies indicate its diagnostic value [21], others report no clear advantage over egg white-sIgE [16,22].

Moreover, predictive values established using the ImmunoCAP system cannot be directly extrapolated to results obtained using other diagnostic platforms due to potential inter-assay variability [23]. Concordance between ImmunoCAP ISAC and the ALEX2 test for milk- and egg-sIgE has been reported to range from 85% to 97.5% [24]. Nevertheless, additional studies are needed to support the use of the ALEX2 platform in diagnosis of CM and HE allergy, as the currently available literature remains scarce, with only one study evaluating HE allergy in children with atopic dermatitis [25].

Attempts have also been made to identify clinical history elements that may help estimate the likelihood of tolerance to milk and baked egg; however, findings have been inconsistent, mainly because of heterogeneity in patient selection criteria across studies [26,27].

In the present study, we evaluated the clinical utility of SPTs to standardized extracts and SPTs to native food allergens, as well as sIgE measurements, including component-resolved diagnostics. Measurements of sIgE were performed using both ImmunoCAP and the ALEX2 multiplex allergy platform. The aim of the study was to improve risk stratification in children with CM or HE allergy and to support more selective referral for OFC testing.

## 2. Materials and Methods

### 2.1. Participants and Study Design

We conducted a retrospective chart review of all patients who underwent OFCs involving baked milk, raw milk, baked egg, and boiled egg at Department of Pediatric Pneumonology and Allergy at Children’s Clinical Hospital UCC MUW from January 2023 to September 2025. The study protocol was approved by the Bioethical Committee of the Medical University of Warsaw (AKBE/158/2026).

Eligibility criteria: All patients who underwent an OFC to baked milk, raw milk, baked egg, or boiled egg in our institution between January 2023 and September 2025 were eligible for inclusion. Inclusion was based solely on the availability of complete clinical documentation, OFC results, and corresponding diagnostic test results (SPT and/or sIgE) performed within the predefined time window prior to OFC.

No formal clinical exclusion criteria were applied for this retrospective analysis, as all consecutive OFCs performed during the study period were considered. Patients were excluded only in cases of incomplete or missing medical records, lack of available pre-challenge diagnostic testing, or insufficient data to determine OFC outcome.

Most subjects had a history of prior allergic reactions to milk (either baked or unheated) or egg (either baked, boiled, or raw). A subset of patients was suspected of having food allergy based on sensitization (positive sIgE results) despite no prior consumption of milk or egg, for example, due to severe atopic dermatitis. Eligibility for OFC was determined by an allergist in accordance with the American Academy of Allergy, Asthma and Immunology (AAAAI)-European Academy of Allergy and Clinical Immunology (EAACI) PRACTALL guidelines [28].

Allergic comorbidities, including asthma, allergic rhinitis, and atopic dermatitis, were not considered exclusion criteria and were documented for statistical analysis.

### 2.2. Diagnostic Testing

Serum sIgE measurements and SPTs performed within six months prior to OFC were considered valid for inclusion in the analysis; when available, results obtained during the same hospital admission as the OFC were preferentially used. In most patients, diagnostic testing was performed during hospitalization prior to OFC, while, in a minority, previously available results were used without repetition, in line with routine clinical practice. Although sensitization profiles may change over time, particularly in young children with evolving food allergy, a six-month interval was considered unlikely to result in clinically meaningful changes in most cases [29].

Serum sIgE concentrations were quantified using the ImmunoCAP system (Uppsala, Sweden), with a sensitization threshold of 0.35 kU/mL, and the ALEX2 platform (MacroArray Diagnostics GmbH, Vienna, Austria), with a sensitization threshold of 0.30 kUA/mL. In all children, the following parameters were assessed using the ImmunoCAP system: sIgE to milk extract, casein, α-lactalbumin, β-lactoglobulin, egg extract, egg white, egg yolk, and total IgE. Assessment of sIgE to egg allergen components by the ImmunoCAP platform, including sIgE measurements to ovomucoid and ovalbumin, was not available at our center during the study period and was therefore not included in the study protocol. Using ALEX2 platform, the following parameters were measured: sIgE to milk extract, casein, α-lactalbumin, β-lactoglobulin, egg white, egg yolk, ovomucoid, ovalbumin, ovotransferrin, lysozyme, serum albumin, and total IgE.

SPT were performed using commercial extracts (Diater, Madrid, Spain), while SPT to native food allergens were performed using fresh products, including fresh milk, raw egg white, and raw egg yolk. A wheal diameter ≥3 mm after 15 min was considered as positive.

### 2.3. Oral Food Challenge Protocol

OFCs were conducted in an open manner in a hospital setting.

All chronic diseases, including asthma and atopic dermatitis, were required to be stable, and no acute conditions or symptoms, such as infections, urticaria, vomiting, or abdominal pain, were permitted at the time of the OFC.

Medications potentially interfering with OFC result were withheld for the recommended periods prior to testing, including antihistamines for at least seven days and systemic corticosteroids for a minimum of two weeks.

Muffins containing either egg or milk were prepared by caregivers according to a standardized recipe. The OFC protocols are shown in Table 1. The doses were increased every 30 min until either an allergic reaction occurred or a full age-appropriate serving was achieved. Challenges were discontinued according to the AAAAI-EAACI PRACTALL stopping criteria, and appropriate treatment was immediately administered by the supervising physician [28].

Allergic reactions occurring during the OFC were assessed according to the World Allergy Organization (WAO) and Błażowski grading scales [30,31]. If no reaction occurred, children were monitored for a minimum of two hours following the completion of the OFC.

### 2.4. Statistical Analysis

All analyses were performed in RStudio 4.3.3. Continuous variables are reported as median with interquartile range (IQR) and categorical variables as frequencies and percentages. Normality was assessed with the Shapiro–Wilk test. Between-group comparisons (OFC-positive vs. OFC-negative) were performed using the Mann–Whitney U test for continuous variables and Fisher’s exact test for categorical variables. A two-tailed *p*-value < 0.05 was considered statistically significant.

Receiver operating characteristic (ROC) curve analysis was used to evaluate the discriminatory performance of individual diagnostic parameters. The area under the ROC curve (AUC) with 95% confidence intervals was calculated using the DeLong method [32]. Parameters with AUC confidence intervals excluding 0.5 were considered to provide statistically significant discrimination. Optimal cut-offs were determined using the Youden index (maximizing sensitivity + specificity − 1) [33]. Additionally, two clinically oriented cut-offs were identified for each parameter: (1) the lowest threshold achieving positive predictive value (PPV) ≥95%, representing a rule-in criterion; and (2) the highest threshold achieving negative predictive value (NPV) ≥90%, representing a rule-out criterion. Bootstrap 95% confidence intervals for all cut-off values were derived from 2000 resampling iterations. Sensitivity, specificity, PPV, and NPV at each threshold were reported with 95% confidence intervals calculated using the Wilson method.

Two-predictor logistic regression models were constructed for each possible pair of parameters within the following categories: SPTs combined with sIgE, two sIgE parameters from the same platform (ImmunoCAP or ALEX2), and sIgE values were log_2_-transformed prior to modeling. Model performance was quantified as the apparent AUC (DeLong, 95% CI) and as the optimism-corrected AUC estimated by the bootstrap Efron-Gong procedure (1000 resampling iterations). Optimism values >0.05 were considered indicative of overfitting. For each model, the maximum predicted OFC+ probability and the number of patients exceeding predicted probability thresholds of 50%, 75%, 90%, and 95% were reported.

Agreement between ALEX2 and ImmunoCAP platforms was assessed for sIgE to components available on both platforms (milk extract and molecular components for milk cohorts; egg white and egg yolk extracts for egg cohorts). Egg cohort platform comparisons were performed on the combined boiled egg and baked egg dataset, and milk cohort comparisons on the combined raw milk and baked milk dataset, on the basis that sensitization status is independent of the form of OFC. Quantitative agreement was assessed by Spearman rank correlation (ρ). Qualitative agreement was assessed by Cohen’s kappa (κ) based on platform-specific sensitization thresholds (≥0.35 kUA/L for ImmunoCAP; ≥0.30 kUA/L for ALEX2, corresponding to the manufacturer’s limit of detection). For different cohorts, predictive performance between platforms was additionally compared using the paired DeLong test, with bootstrap 95% CI for ΔAUC (2000 iterations).

Specific IgE measurements on the ALEX2 platform were reported with a lower limit of detection (LOD) of 0.10 kUA/L; a sensitization threshold of ≥0.30 kUA/L was applied for qualitative classification. For patients with ALEX2 values below the LOD, a value of 0.10 kUA/L was imputed for all quantitative analyses. For ImmunoCAP, the manufacturer-defined sensitization threshold of ≥0.35 kUA/L was used for qualitative classification; however, as this platform reports absolute values below this threshold, all available numerical results including sub-threshold values were retained and used in quantitative statistical analyses.

## 3. Results

### 3.1. Baked Milk Allergy

#### 3.1.1. Patient Characteristics

A total of 42 children of Caucasian ethnicity with suspected CM allergy underwent OFC to baked milk, of whom 13 (31.0%) had a positive challenge (OFC+) and 29 (69.0%) tolerated the baked milk product (OFC−). The two groups did not differ significantly in age, sex and atopic comorbidities distribution, apart from allergic rhinitis, which was more frequent in OFC− than OFC+ patients (65.5% vs. 30.8%; *p* = 0.049) (Table 2). Past anaphylaxis was reported in 53.8% of OFC+ and 44.8% of OFC− patients (*p* = 0.741); a past allergic reaction to milk was reported in 76.9% vs. 55.2% (*p* = 0.303). According to WAO grades, severity of reaction in the 13 OFC+ patients was as follows: grade I (*n* = 1), II (*n* = 8), III (*n* = 3), and IV (*n* = 1); and according to Błażowski grades: I (*n* = 7), II (*n* = 5), and III (*n* = 1). Epinephrine was administered in nine (69.2%) individuals.

#### 3.1.2. Diagnostic Performance of sIgE and SPT in Predicting OFC Outcomes to Baked Milk

The sIgE measurements significantly differentiated OFC outcomes, in contrast to SPT results (Table 3).

Neither results of SPT to raw milk nor SPT to commercial extract significantly differed between OFC+ and OFC− patients. The strongest discriminatory performance was observed for casein- and milk extract-sIgE measured by ImmunoCAP, with markedly higher concentrations in OFC+ patients (median 13.64 vs. 3.04 kUA/L, *p* = 0.001 for casein-sIgE; 20.55 vs. 5.60 kUA/L, *p* = 0.007 for milk extract-sIgE). Total IgE levels did not differ between groups, and normalization sIgE to total IgE did not improve discrimination (Table S1).

Consistently, analysis of predictive performance (Table 4) confirmed that casein- and milk extracts-sIgE showed the highest diagnostic accuracy. Casein-sIgE achieved the highest AUC (0.842, 95% CI 0.709–0.975), with an optimal cut-off of 5.27 kUA/L (100% sensitivity, 62% specificity; PPV 53%, NPV 100%). The PPV ≥95% threshold was 22.14 kUA/L, while the NPV ≥90% cut-off was 7.44 kUA/L. Milk extract-sIgE demonstrated slightly lower but still good performance (AUC 0.805, 95% CI 0.651–0.959), with an optimal cut-off of 6.18 kUA/L (100% sensitivity, 57% specificity; PPV 53%, NPV 100%). The NPV ≥90% threshold was 7.59 kUA/L, whereas a PPV ≥95% cut-off could not be established. Although several ALEX2-derived component-sIgE measurements differed significantly between OFC+ and OFC− patients, the overall diagnostic performance of the ALEX2 platform was inferior to that of ImmunoCAP.

#### 3.1.3. Two-Predictor Logistic Regression Models

Two-predictor logistic regression models combining two predictors showed improved discriminative ability compared with single markers, with optimism-corrected AUC values ranging from 0.59 to 0.89. The best-performing models consistently involved ImmunoCAP-derived casein-sIgE, often in combination with either sIgE to other components or SPT results. These combined models achieved higher AUCs than individual tests, suggesting additive diagnostic value when integrating complementary parameters. However, the improvement was moderate (Table S2).

### 3.2. Raw Milk Allergy

#### 3.2.1. Patient Characteristics

A total of 43 children of Caucasian ethnicity with suspected raw milk allergy were included, of whom 18 (41.9%) had an OFC+ and 25 (58.1%) an OFC−. The two groups did not differ significantly in age, sex distribution, or comorbid atopic conditions. Tolerance to baked milk was documented in 88.2% of OFC+ and 88.8% of OFC− patients (*p* > 0.999), indicating that the study cohort was dominated by the baked-milk-tolerant phenotype. A past allergic reaction to raw milk was reported more frequently in the OFC+ group (66.7% vs. 40.0%; *p* = 0.124), while a recent allergic reaction within one year was observed exclusively in the OFC− group (16.7% vs. 0%; *p* = 0.122); however, results did not reach statistical significance. Past anaphylaxis was reported in 33.3% of OFC+ and 44.0% of OFC− patients (*p* = 0.530). According to WAO grades, severity of reaction in the 18 OFC+ patients was as follows: grade I (*n* = 8), II (*n* = 9), and III (*n* = 1); and according to Błażowski grades: I (*n* = 9) and II (*n* = 9). Epinephrine was administered in five (27.8%) individuals. Patient characteristics are summarized in Table 5.

#### 3.2.2. Diagnostic Performance of sIgE and SPT in Predicting OFC Outcomes to Raw Milk

Both sIgE and SPT contributed to differentiating OFC outcomes to raw milk, although their diagnostic utility varied (Table 6). In contrast to baked milk, results of SPT to raw milk showed a significant difference between OFC+ and OFC− patients, with larger wheal diameters observed in the OFC+ group, while results of SPT to commercial extract did not differentiate between groups.

Among the ImmunoCAP parameters, the results of milk extract-sIgE and α-lactalbumin-sIgE differed significantly between OFC+ and OFC− patients, with higher levels observed in the OFC+ group. In contrast, differences in results of β-lactoglobulin- and casein-sIgE did not reach statistical significance. On the ALEX2 platform, only results of β-lactoglobulin-sIgE showed a significant difference between groups, while the differences in results of sIgE to other components demonstrated only non-significant trends. Total IgE levels did not differ significantly between groups, and normalization sIgE to total IgE did not improve discrimination (Table S3).

Consistently, analysis of predictive performance (Table 7) indicated moderate diagnostic accuracy of the evaluated markers. The highest AUC values were observed for β-lactoglobulin-sIgE measured by ALEX2 (AUC 0.733, 95% CI 0.548–0.918) and α-lactalbumin-sIgE measured by ImmunoCAP (AUC 0.726, 95% CI 0.550–0.903), followed by milk extract-sIgE assessed by ImmunoCAP (AUC 0.714, 95% CI 0.541–0.888). SPT to raw milk showed slightly lower performance (AUC 0.692, 95% CI 0.527–0.857).

Optimal Youden cut-offs were identified at 0.28 kUA/L for β-lactoglobulin-sIgE (ALEX2), 2.83 kUA/L for α-lactalbumin-sIgE, 3.03 kUA/L for milk extract-sIgE (both ImmunoCAP), and 10 mm for SPT to raw milk. α-lactalbumin-sIgE demonstrated the highest specificity (90%) and positive likelihood ratio (LR+ 5.91), supporting its potential as a rule-in parameter. Milk extract-sIgE showed a more balanced sensitivity and specificity profile (81% and 71%, respectively), while β-lactoglobulin-sIgE (ALEX2) was characterized by high sensitivity (85%) but lower specificity (58%). SPT to raw milk, despite relatively high specificity (84%), had limited sensitivity (47%).

High rule-in thresholds (PPV ≥ 95%) were identified for β-lactoglobulin-sIgE (ALEX2; 5.49 kUA/L), milk extract-sIgE (24.55 kUA/L), and SPT to raw milk (13.5 mm), whereas rule-out thresholds (NPV ≥ 90%) were achieved only for α-lactalbumin-sIgE and milk extract-sIgE at low levels.

#### 3.2.3. Two-Predictor Logistic Regression Models

Two-predictor logistic regression models showed modest improvement over single parameters, with optimism-corrected AUC values ranging from 0.54 to 0.73. The best-performing models consistently included β-lactoglobulin-sIgE as a key component-sIgE, particularly in combination with other sIgE markers (e.g., α-lactalbumin or milk extract on ALEX2) or with SPT to raw milk. Models combining two ALEX2-derived components-sIgE ranked highest, suggesting complementary value of paired whey-protein sensitization profiles. However, overall discrimination remained moderate, and no model achieved a predicted probability ≥0.95, indicating that none could reliably replace OFC (Table S4).

### 3.3. Agreement Between ALEX2 and ImmunoCAP Platforms—Milk Cohorts

**Figure 1:**
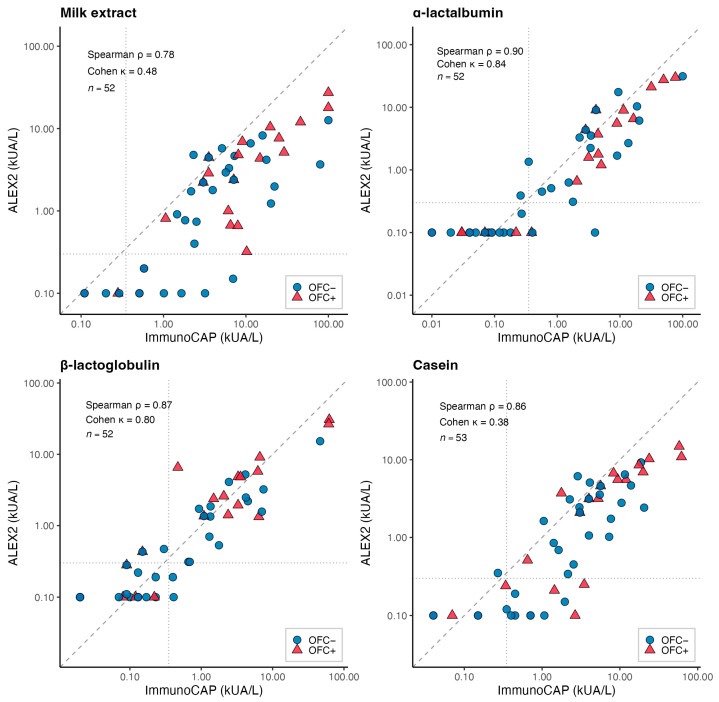
Quantitative and qualitative agreement between ALEX2 and ImmunoCAP for cow’s milk extract-sIgEs and molecular components (α-lactalbumin, β-lactoglobulin, and casein)-sIgEs in the combined cohort (baked milk + raw milk, *n* = 52–53). Each point represents one patient, colored by OFC outcome (OFC+: red, OFC−: blue). Axes are log-scaled. Dotted lines indicate platform-specific sensitization thresholds (0.35 kUA/L for ImmunoCAP, 0.30 kUA/L for ALEX2). The dashed diagonal represents the line of perfect agreement. Spearman rho and Cohen’s kappa values are shown in each panel.

Platform agreement was assessed in the combined raw milk and baked milk cohort (*n* = 85; 52–53 patients with paired measurements per component; Figure 1, Table S5). Quantitative agreement was strong for all four components (Spearman ρ = 0.783–0.899; all *p* < 0.001). The results of α-lactalbumin-sIgE and β-lactoglobulin-sIgE levels showed almost perfect agreement (κ = 0.840 and κ = 0.802, respectively; concordance 92.3% and 90.4%), with only minimal discordance between platforms. In contrast, agreement for results of casein-sIgEs was only moderate (κ = 0.385; concordance 77.4%), with 11 patients classified as sensitized on ImmunoCAP but not on ALEX2 and only 1 in the opposite direction—indicating systematic ALEX2 underestimation of casein sensitization near the threshold. The results of milk extract-sIgEs showed intermediate qualitative agreement (κ = 0.484; concordance 84.6%), with all eight discordant cases again attributable to ImmunoCAP-only classification.

Predictive performance of each parameter for OFC outcome did not differ significantly between platforms in either cohort (Tables S6 and S7). In the raw milk cohort, ΔAUC ranged from −0.053 to +0.091 across components-sIgEs (all *p* ≥ 0.18). In the baked milk cohort, ΔAUC ranged from −0.008 to +0.082 (all *p* ≥ 0.24), with casein-sIgE showing numerically higher AUC on ALEX2 (0.912 vs. 0.853; *p* = 0.297) but without statistical significance. All confidence intervals for ΔAUC included zero.

### 3.4. Boiled Egg Allergy

#### 3.4.1. Patient Characteristics

A total of 54 children of Caucasian ethnicity underwent OFC to boiled egg; 18 (33.3%) had an OFC+ and 36 (66.7%) tolerated boiled egg. The groups were comparable in age, sex, and all atopic comorbidities. Tolerance to baked egg was present in 77.8% of OFC+ and 88.9% of OFC− patients (*p* = 0.418), confirming the predominantly baked-egg-tolerant phenotype. No patient experienced an allergic reaction to egg in the preceding year. Patient characteristics are summarized in Table 8. Past anaphylaxis was reported in 33.3% of OFC+ and 30.6% of OFC− patients (*p* > 0.999); a past allergic reaction to egg was reported in 50.0% vs. 52.8% (*p* > 0.999). According to WAO grades, severity of reaction in the 18 OFC+ patients was as follows: grade I (*n* = 6), II (*n* = 10), and III (*n* = 2); and according to Błażowski grades: I (*n* = 10), II (*n* = 7), and III (*n* = 1). Epinephrine was administered in 15 (83.3%) individuals.

#### 3.4.2. Diagnostic Performance of sIgE and SPT in Predicting OFC Outcomes to Boiled Egg

All methods—SPT to commercial extracts, SPT to raw egg white, raw egg yolk, and sIgE measurements—contributed to differentiation of OFC outcomes to boiled egg, although their relative performance varied by method and allergen (Table 9). Significant differences between OFC+ and OFC− patients showed SPTs to raw egg white, raw egg yolk, and SPTs to commercial egg yolk extract, whereas SPT to commercial egg white extract did not discriminate between groups.

ImmunoCAP measurements demonstrated consistent but non-significant trends toward higher sIgE levels to different allergens in OFC+ patients for egg extract, egg white, and egg yolk. In contrast, ALEX2 measurements showed significant differences for sIgE levels to multiple components, including egg white extract, ovomucoid, egg yolk extract, and ovalbumin. The results of sIgE measurements to such components as lysozyme and serum albumin were excluded due to a high proportion of non-informative results. Ratio of tIgE to sIgE did not improve discrimination between the groups (Table S8).

Consistently, analysis of predictive performance (Table 10) confirmed moderate diagnostic accuracy across parameters, with seven out of twelve showing statistically significant discrimination. The highest AUC values were observed for SPT to raw egg yolk (AUC 0.738, 95% CI 0.586–0.890), egg white extract-sIgE measured by ALEX2 (AUC 0.735, 95% CI 0.576–0.894), SPT to raw egg white (AUC 0.725, 95% CI 0.580–0.870), and ovomucoid-sIgE (ALEX2; AUC 0.713, 95% CI 0.543–0.882). Additional parameters, including egg yolk extract-sIgE (AUC 0.709), ovalbumin-sIgE (AUC 0.698), and SPT to commercial egg yolk extract (AUC 0.681), showed slightly lower but acceptable performance.

For clinical rule-in (PPV ≥ 95%), high cut-off values were identified exclusively for ALEX2-derived sIgE parameters, including egg white extract-sIgE, ovomucoid-sIgE, and ovalbumin-sIgE (all approximately ≥15.7–15.9 kUA/L). In contrast, rule-out thresholds (NPV ≥90%) were achieved only for SPT parameters, specifically for raw egg white (≤6.5 mm) and raw egg yolk (≤1.0 mm). Overall, these findings suggest complementary roles of SPT and sIgE, with ALEX2-derived component testing providing the strongest rule-in capacity, and SPT offering limited but clinically relevant rule-out utility.

#### 3.4.3. Two-Predictor Logistic Regression Models

In contrast, two-predictor logistic regression models demonstrated modest improvements in discrimination compared with single parameters, with the best-performing models combining SPTs and ALEX2-derived sIgE measurements. The top model (SPT to commercial egg white extract + sIgE to egg white extract measured by ALEX2 platform) achieved an optimism-corrected AUC of 0.778, representing a small gain over the best single predictor. Similar incremental improvements were observed for models combining SPT to egg yolk extract or ovomucoid-sIgE. However, despite these gains, overall model performance remained moderate, and no combination reliably identified patients with a predicted probability of OFC positivity ≥ 95% (Table S9).

### 3.5. Baked Egg Allergy

#### 3.5.1. Patient Characteristics

A total of 54 children of Caucasian ethnicity underwent OFC to baked egg; 15 (27.8%) had an OFC+ and 39 (72.2%) tolerated baked egg. The groups did not differ significantly in age, sex or prevalence of atopic comorbidities. A past allergic reaction to egg was reported in 73.3% of OFC+ and 61.5% of OFC− patients (*p* = 0.532). Past anaphylaxis was significantly more frequent in OFC+ than OFC− patients (60.0% vs. 17.9%; *p* = 0.006). An allergic reaction to egg within the preceding year was reported in 6.7% of OFC+ and 2.6% of OFC− patients (*p* = 0.482). Patient characteristics are summarized in Table 11. According to WAO grade, severity of reaction in the 15 OFC+ patients was as follows: grade I (*n* = 4), II (*n* = 10), and III (*n* = 1); and according to Błażowski grades: I (*n* = 7), II (*n* = 8), and III (*n* = 1). Epinephrine was administered in four (25%) individuals.

#### 3.5.2. Diagnostic Performance of sIgE and SPT in Predicting OFC Outcomes to Baked Egg

Both SPTs and sIgE measurements contributed to differentiation of OFC outcomes to baked egg, although their performance varied by method (Table 12). Among SPT parameters, only SPT to commercial egg white extract significantly distinguished OFC+ from OFC− patients, while SPT to raw egg white and raw egg yolk showed only borderline trends, and SPT to egg yolk extract was not discriminatory.

ImmunoCAP measurements did not reach statistical significance for any parameter, despite consistently higher sIgE levels to various components in OFC+ patients. In contrast, the analysis of sIgE results measured by ALEX2 platform showed significant differences for levels of ovalbumin-sIgE, ovotransferrin-sIgE, egg white extract-sIgE, and egg yolk extract-sIgE, while differences in levels of ovomucoid-sIgE showed a non-significant trend. The results of lysozyme-sIgE and serum albumin-sIgE were excluded from the analysis due to a high proportion of non-informative results. Total IgE levels did not differ between groups, and normalization sIgE to total IgE did not improve discrimination (Table S10).

Analysis of predictive performance (Table 13) confirmed moderate to good diagnostic accuracy, with six of twelve parameters showing significant discrimination. The strongest predictor was sIgE to ovalbumin measured by ALEX2 (AUC 0.853, 95% CI 0.718–0.988), followed by sIgE to ovotransferrin (AUC 0.772, 95%CI 0.568–0.977), SPT to white extract (AUC 0.762, 95%CI 0.602–0.922), and sIgE to egg white extract in ALEX2 (AUC 0.754, 95%CI 0.558–0.950). SPT to raw egg yolk (AUC 0.719, 95%CI 0.553–0.884) and sIgE to egg yolk extract ALEX2 (AUC 0.736, 95%CI 0.555–0.916) provided additional significant but lower discrimination.

For clinical rule-in (PPV ≥ 95%), cut-offs were identified for three parameters. The optimal cut-off for egg white extract-sIgE measured using the ALEX2 platform was ≥26.27 kUA/L, corresponding to a PPV of ≥95%. The cut-off value for the size of SPT to raw egg yolk of ≥23.0 mm achieved a PPV of ≥95%. Similarly, an SPT wheal diameter of ≥15.5 mm for commercial egg white extract achieved a PPV of ≥95%. In contrast, ovalbumin-sIgE measured using the ALEX2 platform did not achieve a PPV of ≥95% at any threshold despite its high AUC, as both OFC-positive and OFC-negative groups included patients with very high values, precluding the identification of a purely discriminatory cut-off.

For clinical rule-out (NPV ≥ 90%), cut-off values were established for six parameters, including ovalbumin-sIgE (≤9.25 kUA/L), egg white extract-sIgE (≤9.01 kUA/L), egg yolk extract-sIgE (≤0.61 kUA/L; specificity 52%), ovotransferrin-sIgE (≤4.50 kUA/L), SPT to commercial egg white extract (≤5.5 mm), and SPT to raw egg yolk (≤5.5 mm).

#### 3.5.3. Two-Predictor Logistic Regression Models

The best-performing multivariable model combined ALEX2-derived ovomucoid-sIgE and ovalbumin-sIgE (corrected AUC = 0.848) but did not improve upon the performance of ovalbumin-sIgE alone (AUC = 0.853). No patient reached a predicted OFC+ probability ≥90%, indicating that the molecular sensitization profile alone was insufficient to identify high-risk individuals with clinical certainty. All top models exclusively used ALEX2 components (Table S11).

### 3.6. Agreement Between ALEX2 and ImmunoCAP Platforms

**Figure 2:**
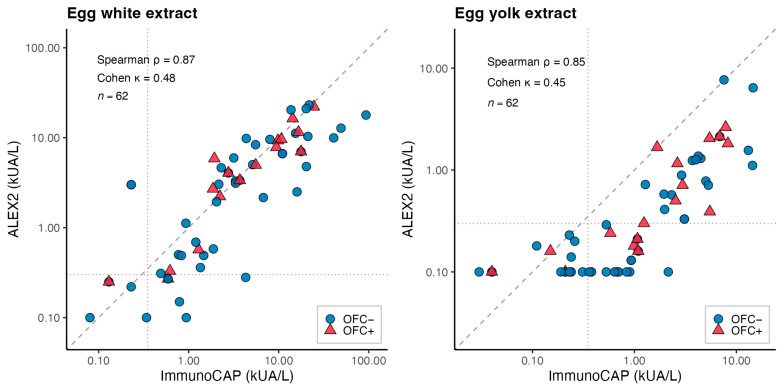
Quantitative and qualitative agreement between ALEX2 and ImmunoCAP platforms for results of egg white extract-sIgE and egg yolk extract-sIgE in the combined cohort (baked egg + boiled egg, *n* = 62). Each point represents one patient, colored by OFC outcome (OFC+: red, OFC−: blue). Axes are log-scaled. Dotted lines indicate platform-specific sensitization thresholds (0.35 kUA/L for ImmunoCAP, 0.30 kUA/L for ALEX2). The dashed diagonal represents the line of perfect agreement. Spearman rho and Cohen’s kappa values are shown in each panel.

Platform agreement was assessed in the combined boiled and baked egg cohort (n = 108; 62 patients with paired measurements) (Table S12, Figure 2). Quantitative agreement between the ALEX2 and ImmunoCAP platforms was strong for both allergen extract-sIgE measurements (Spearman ρ = 0.874 for egg white extract-sIgE and ρ = 0.853 for egg yolk extract-sIgE; both *p* < 0.001). In contrast, qualitative agreement was only moderate, with κ = 0.484 for egg white extract-sIgE (87.1% concordance) and κ = 0.445 for egg yolk extract-sIgE (71.0% concordance). A clear asymmetry was observed, with more sensitizations detected by ImmunoCAP, particularly for egg yolk extract-sIgE.

Despite these differences, predictive performance of each parameter for OFC outcomes did not differ significantly between platforms in either boiled or baked egg cohorts, with small and non-significant ΔAUC values and confidence intervals including zero. The results of sIgE to molecular components (ovomucoid, ovalbumin, and ovotransferrin) were assessed only by ALEX2 and were not included in the platform comparison (Tables S13 and S14).

## 4. Discussion

The present study corroborates and extends previous meta-analytical evidence regarding the diagnostic accuracy of tests for CM and HE allergy [14]. In addition, our findings provide further insight into phenotype-specific differences and the comparative performance of various diagnostic approaches. A strength of our study is the broad range of diagnostic methods performed, including SPT, sIgE measurements, and molecular diagnostics using the ALEX2 platform and ImmunoCAP platform, which enabled a more comprehensive assessment of sensitization profiles. Our results suggest that, although SPT and sIgE testing remain useful tools in the diagnostic workup of CM and HE allergy, their diagnostic performance is limited and should be interpreted in the context of the overall clinical picture.

**Cow’s Milk Allergy**

**Baked Milk Allergy**

Recent evidence regarding predictors of baked milk tolerance remains heterogeneous. In the most recently published systematic review, meta-analysis for baked milk allergy could not be performed because of insufficient reporting of diagnostic performance measures across available studies [14]. Existing studies have yielded inconsistent results, ranging from a lack of statistically significant differences for results of SPT and sIgE measurements directed against milk extracts and molecular components [34,35] to reports demonstrating significant associations, particularly for casein-sIgE [36,37]. One study additionally identified superior predictive performance for SPT to raw milk [38].

In our cohort, neither SPT to commercial extract nor SPT to raw milk significantly differentiated OFC+ from OFC− patients, whereas casein-sIgE demonstrated the strongest predictive performance. Specifically, casein-sIgE measured by ImmunoCAP achieved the highest AUC (0.842, 95% CI 0.709–0.975), with an optimal cut-off value of 5.27 kUA/L, corresponding to 100% sensitivity and 62% specificity (PPV 53%, NPV 100%). Furthermore, multivariable models incorporating casein-sIgE together with additional sIgE to other components and SPT results further improved predictive performance, with optimism-corrected AUC values reaching approximately 0.86–0.89, comparable to or slightly exceeding values reported in previous individual studies. A comparable diagnostic performance was observed for casein-sIgE measured using the ALEX2 platform, which yielded a similar optimal cut-off value of 5.56 kUA/L. However, the sensitivity at this threshold was lower (80%), indicating a reduced ability to identify all patients with baked milk allergy despite broadly similar overall discriminative performance.

Importantly, these findings are consistent with previous studies conducted in patients intolerant to raw milk who subsequently underwent baked milk OFCs [39]. In that context, the optimal cut-off for casein-sIgE was reported as 4.68 kUA/L, with 75% sensitivity, 84% specificity, and an AUC of 0.827 [18]. The close concordance between these thresholds and AUC estimates supports the robustness and reproducibility of casein-sIgE as a key biomarker of baked milk reactivity across different clinical populations and study designs [19,40].

**Raw Milk Allergy**

Prediction of raw milk allergy was less accurate, with AUC values ranging from approximately 0.60 to 0.73 across SPT to commercial extract, SPT to raw milk and sIgE measurements, including component-resolved diagnostics. In the recent meta-analysis [14], SPT to raw milk demonstrated consistently higher sensitivity and specificity compared with SPT to commercial extract, which is in line with our findings, where SPT to raw milk also showed superior discriminative performance.

In our cohort, whey proteins-sIgE—particularly β-lactoglobulin-sIgE measured using the ALEX2 platform and α-lactalbumin-sIgE measured using ImmunoCAP—demonstrated greater diagnostic utility for predicting reactivity to raw milk, whereas casein-sIgE appeared to play a less prominent role. In contrast, in the studies included in the meta-analysis, casein-sIgE showed higher diagnostic performance, which differs from our observations. Importantly, the meta-analytic data did not provide information on tolerance to baked milk, which limits direct comparability with our cohort. Our study population was highly enriched for patients tolerant to baked milk (approximately 88%), indicating a predominance of a baked-milk-tolerant phenotype. Based on our findings, we hypothesize that this subgroup may exhibit distinct sensitization patterns, in which IgE responses to heat-labile whey proteins (e.g., α-lactalbumin and β-lactoglobulin) appear to be more closely associated with clinical reactivity to raw milk, whereas sensitization to heat-stable proteins such as casein has more frequently been linked to persistent milk allergy and reactivity to baked milk. Accordingly, our findings may be consistent with previous observations suggesting that sensitization to whey proteins could play a greater role in reactions to unheated milk among baked-milk-tolerant individuals, while casein sensitization is more commonly associated with more persistent phenotypes [41,42,43]. However, this apparent difference should be interpreted with caution and should not be considered evidence of distinct underlying pathogenic mechanisms. The observed distribution of component relevance most likely reflects both the clinical characteristics of the study population and the sequential approach applied during OFCs. Patients undergoing raw milk OFCs constituted a pre-selected subgroup, as children reacting to baked milk are generally considered highly likely to react to raw milk and therefore are less frequently referred for subsequent raw milk challenges. Consequently, the raw milk cohort was enriched for individuals with a lower baseline probability of severe milk allergy and a higher prevalence of baked milk tolerance. This selective referral process may have influenced the relative diagnostic performance of individual milk components and potentially affected the estimated AUC values and optimal cut-off points. Had patients reactive to baked milk been systematically included in the raw milk analysis, the association between casein sensitization and challenge outcomes would likely have been stronger. Therefore, the present findings should be interpreted within the context of the clinical decision-making pathway and patient selection process rather than as evidence of a fundamentally different hierarchy of clinically relevant milk allergens.

**Hen’s Egg Allergy**

In HE allergy, our findings are generally consistent with previously published meta-analyses [14], although direct comparison is limited by important methodological differences.

Most previous studies evaluating the diagnostic performance of egg allergen components have relied on component-resolved diagnostics performed using the ImmunoCAP platform, particularly measurements of ovomucoid- and ovalbumin-sIgE. In contrast, in our study, component-resolved diagnostics for egg allergy were assessed exclusively using the ALEX2 platform. Measurements of egg allergen components-sIgE using ImmunoCAP, including ovomucoid-sIgE and ovalbumin-sIgE, were not available at our center during the study period and therefore could not be incorporated into the study protocol. Consequently, only egg white-, egg yolk-, and egg extract-sIgE concentrations were measured using ImmunoCAP. This limitation precluded a direct comparison of the diagnostic performance of individual sIgE to egg allergen components between the two platforms and restricted our assessment of inter-platform concordance largely to extract-level measurements.

**Boiled Egg Allergy**

In the boiled egg cohort, SPTs, either to commercial extract or native products, again demonstrated only moderate diagnostic utility, with the highest diagnostic performance observed for SPT to raw egg white and raw egg yolk. Similarly, ImmunoCAP-based measurements, i.e., egg white extract-sIgE and egg yolk extract-sIgE, showed moderate accuracy and did not consistently reach statistical significance, despite a trend toward higher values in OFC+ patients. These findings are consistent with a recent report demonstrating only moderate discriminatory performance for both SPTs and egg white extract-sIgE and egg yolk extract-sIgE (AUC approximately 0.77–0.78 for key parameters), without identification of a single dominant predictor [44,45]. Corresponding measurements performed using the ALEX2 platform demonstrated somewhat better diagnostic performance, particularly for egg white extract-sIgE; however, their overall discriminative ability remained only moderate and was insufficient to support their use as standalone predictors of OFC outcomes.

Within component-resolved diagnostics, ovomucoid-sIgE measured using ALEX2 platform demonstrated the highest diagnostic value for boiled egg allergy, which is consistent with previous reports [45]. These findings suggest a substantial degree of agreement between the ImmunoCAP and ALEX2 platforms.

**Baked Egg Allergy**

According to recent reports, SPTs have limited utility in predicting baked egg allergy [46]. In our study, only SPT to egg white extract demonstrated sufficient discriminative ability to distinguish children with baked egg allergy from those tolerant to baked egg. This observation is consistent with a previously published meta-analysis [14], in which the diagnostic accuracy of SPT to egg white was also reported to be limited.

Similarly to the findings in the boiled egg cohort, ImmunoCAP-based measurements of egg white extract-sIgE and egg yolk extract-sIgE did not significantly differentiate children with baked egg allergy from those who tolerated baked egg. Among the ALEX2-derived sIgE measurements, egg white extract-sIgE showed the highest diagnostic performance, with an AUC of 0.754 (95% CI 0.558–0.950). The optimal cut-off value was 9.29kUA/L, corresponding to a sensitivity of 88%, specificity of 65%, PPV of 39%, and NPV of 95%. Overall, these findings suggest broadly similar diagnostic patterns across the ImmunoCAP and ALEX2 platforms and are in line with previously published reports. In a recent meta-analysis, egg white-sIgE measured using the ImmunoCAP platform demonstrated high specificity (94%) but relatively low sensitivity (40%) for predicting baked egg allergy at a cut-off value of 8 kUA/L, although the reported thresholds varied considerably, ranging from 6 to 50 kUA/L.

With regard to component-resolved diagnostics assessed using the ALEX2 platform, ovalbumin-sIgE demonstrated the highest diagnostic value for distinguishing children with baked HE allergy. In our study, ovomucoid-sIgE did not reach statistical significance, despite showing a trend toward improved discriminatory performance, which may be related to limited sample size and the high degree of overall sensitization within the cohort. However, our findings are consistent with previous reports indicating that the predictive value of ovomucoid-sIgE for OFC outcomes in baked egg allergy remains controversial, as some studies have demonstrated its diagnostic utility [21,47], whereas others have found no significant superiority compared with egg white extract-sIgE [16,22,48]. Additionally, in the study by Krawiec et al. [16], both ovalbumin-sIgE and ovomucoid-sIgE demonstrated comparable diagnostic performance for baked egg allergy, without clear superiority of either component. In that study, the AUC values for ovomucoid-sIgE and ovalbumin-sIgE were similar, suggesting largely overlapping diagnostic information rather than a distinct advantage of ovomucoid-sIgE despite its higher thermostability. Notably, in the study by Krawiec et al. [16], ImmunoCAP-based assessment of ovalbumin-sIgE demonstrated lower predictive performance compared with the ALEX2-derived ovalbumin-sIgE obtained in our cohort, further supporting the possibility of assay-dependent differences in the diagnostic value of component-resolved testing.

**Combined two-predictor logistic regression**

Combined two-predictor logistic regression models for parameters of baked and raw milk allergy, as well as baked and boiled egg allergy, provided only modest improvements over single predictors, with limited gains in AUC values and no clinically meaningful increase in discriminatory performance. Importantly, none of the models achieved sufficiently high predicted probabilities to reliably replace OFCs, indicating that the combination of diagnostic markers did not substantially enhance overall diagnostic utility.

**Comparison between ImmunoCAP and ALEX2 platforms**

An important advantage of our study is the direct comparison of two diagnostic platforms, namely ImmunoCAP and ALEX2. Overall, ALEX2 demonstrated good concordance with ImmunoCAP, supporting its potential utility in clinical practice. However, systematic differences in absolute values were observed, including consistently lower measurements for milk extract and casein obtained using the ALEX2 platform. The comparison of ALEX2 and ImmunoCAP results in the present study also highlights important considerations regarding the interpretation of multiplex and singleplex sIgE assays. The comparison of ALEX2 and ImmunoCAP results in the present study provides additional insight into the interpretation of different sIgE testing approaches. ALEX2 enables the simultaneous assessment of a broad range of allergen extracts and molecular components within a single assay, thereby providing a comprehensive characterization of individual sensitization profiles. This may facilitate the identification of clinically relevant sensitization patterns and support a more detailed assessment of food allergy phenotypes. In contrast, ImmunoCAP is typically used to quantify selected allergen-specific IgE responses individually and remains the most extensively validated method in routine allergy diagnostics. Although the overall diagnostic performance of ALEX2 and ImmunoCAP was broadly comparable in our cohort, quantitative differences were observed for some allergens. These discrepancies may reflect differences in allergen composition, calibration procedures, and other analytical characteristics of the assays. Therefore, sIgE results should be interpreted within the context of the specific assay used, and diagnostic thresholds established for one method should not be directly extrapolated to another without appropriate validation. Despite these quantitative discrepancies, overall diagnostic performance remained comparable between the two methods, suggesting that clinical interpretation is broadly consistent, although platform-specific cut-off values are required.

These findings are in line with previous studies demonstrating high overall agreement and good quantitative correlation between ALEX2 and ImmunoCAP, including food allergens such as milk and egg components [24,49]. At the same time, prior reports have emphasized that the two methods are not directly interchangeable due to differences in absolute IgE values obtained with each assay.

Importantly, in our study, even after combining multiple predictors in logistic regression models, none of the evaluated approaches achieved sufficient diagnostic accuracy to replace OFCs. The best-performing models did not reach probability thresholds that would justify omission of OFC, which is consistent with the broader literature highlighting the limitations of currently available biomarkers of food allergy. Furthermore, the relatively small sample size limits the stability and generalizability of our findings, particularly with regard to ROC-derived cut-off values and multivariable modeling.

**Clinical implications**

Although no single biomarker was sufficiently accurate to replace OFCs, the present findings suggest that the combined assessment of SPT results, extract-specific IgE, and allergen component sensitization patterns may support more informed pre-challenge risk stratification. Such information may assist clinicians in selecting patients most likely to benefit from OFC, prioritizing referrals, and planning the appropriate level of supervision during challenge procedures.

An additional strength of this study is the evaluation of the ALEX2 multiplex platform in relation to clinically verified OFC outcomes. Our results indicate that ALEX2 provides diagnostically relevant information that is broadly comparable to that obtained with ImmunoCAP for many evaluated parameters, while simultaneously enabling comprehensive assessment of multiple allergen components within a single assay. At the same time, the observed differences between platforms, particularly for selected allergens such as casein, highlight the importance of platform-specific interpretation of sIgE results and caution against the direct transfer of diagnostic thresholds between assays. Further prospective studies are needed to validate ALEX2-derived cut-off values and to define its role in routine clinical decision-making regarding food challenges.

**Limitations of the study**

Several limitations of this study should be acknowledged.

First, the diagnostic performance of the evaluated biomarkers for HE and CM allergy was generally moderate, as reflected by the observed AUC values and, for several parameters, wide confidence intervals resulting from the limited sample size. The study was also not designed or powered a priori to test the primary endpoints with a pre-specified statistical power. Consequently, all proposed cut-off values should be regarded as exploratory estimates rather than validated clinical decision thresholds. Therefore, none of the proposed cut-off values should be applied in clinical practice without prior validation in larger, independent cohorts, ideally including patients with diverse clinical phenotypes and assessed across different diagnostic platforms.

Particular caution is warranted when interpreting the proposed cut-off values for egg allergen components, as component-resolved diagnostics for egg allergy were available exclusively through the ALEX2 platform in the present study. Owing to known methodological differences between ALEX2 and ImmunoCAP, including differences in allergen composition, calibration procedures, and analytical characteristics, these thresholds should not be directly extrapolated to ImmunoCAP-based measurements and require independent validation across multiple diagnostic platforms.

Second, the comparison between the ALEX2 and ImmunoCAP platforms in the egg allergy cohort was limited by the absence of ImmunoCAP measurements for key egg allergen components, particularly ovomucoid and ovalbumin. As these components are among the most clinically relevant biomarkers for predicting egg allergy persistence and challenge outcomes, their unavailability restricted the component-level comparison between platforms and limited conclusions regarding inter-platform concordance to extract-based measurements. Consequently, the diagnostic performance of ImmunoCAP in the egg cohorts may have been underestimated.

Third, the study population may have been affected by spectrum bias, as patients referred for OFCs represented a clinically pre-selected group rather than an unselected population of food-allergic children. This selection process may have influenced estimates of diagnostic accuracy, including AUC values and proposed cut-off points. This limitation is inherent to the retrospective design of the study, as participant selection was based on routine clinical decision-making rather than predefined research criteria. Nevertheless, despite the potential influence of spectrum bias, the main findings of our study are broadly consistent with previously published evidence regarding the diagnostic utility of skin prick testing, allergen-specific IgE measurements, and component-resolved diagnostics in predicting food challenge outcomes. This concordance supports the overall validity of our observations, although the reported diagnostic performance estimates and cut-off values should be interpreted with appropriate caution.

Fourth, all OFCss were performed as open challenges rather than double-blind, placebo-controlled food challenges. Although open OFCs reflect routine clinical practice and are widely used in the assessment of food allergy, they may be more susceptible to observer and expectation bias, particularly when subjective symptoms are reported.

An additional limitation is that SPT and sIgE measurements performed up to six months before OFC were considered eligible for analysis. Although this approach reflects routine clinical practice, temporal changes in sensitization profiles may have introduced measurement variability and potentially attenuated the observed associations between diagnostic test results and OFC outcomes.

Finally, as this was a single-center study, the findings may not be fully generalizable to other populations and clinical settings. Larger, multicenter studies incorporating a comprehensive component-resolved diagnostic approach on both platforms are needed to validate our findings and establish clinically applicable cutoff values.

## 5. Conclusions

Skin prick testing, serum-specific IgE measurements, and component-resolved diagnostics demonstrated variable diagnostic performance in predicting the outcomes of OFCs to baked and raw cow’s milk, as well as baked and boiled hen’s egg. Among the evaluated methods, SPTs performed with native food allergens generally showed the highest discriminative capacity, particularly in the assessment of raw milk and boiled egg allergy, whereas conventional extract-based SPTs demonstrated more limited diagnostic utility. Component-resolved diagnostics provided additional information beyond extract-based testing, although the diagnostic relevance of individual allergen components-sIgEs varied according to the clinical phenotype and diagnostic platform used.

In cow’s milk allergy, casein-sIgE appeared to be more informative in the assessment of baked milk allergy, whereas sensitization to α-lactalbumin and β-lactoglobulin showed a stronger association with raw milk challenge outcomes in our study population. However, this observation may, at least in part, reflect the clinical selection of patients undergoing raw milk challenges, resulting in a cohort enriched for baked-milk-–tolerant individuals rather than the full spectrum of cow’s milk allergy phenotypes. In hen’s egg allergy, ovomucoid-sIgE demonstrated the highest diagnostic performance for boiled egg allergy, while ovalbumin-sIgE showed the best performance for baked egg allergy when assessed using the ALEX2 platform. However, these findings should be interpreted cautiously, as component-resolved diagnostics for egg allergy were available only through ALEX2, limiting direct comparison with ImmunoCAP-based measurements.

Overall, ALEX2 showed good agreement with ImmunoCAP for most evaluated parameters, although systematic differences were observed for selected allergens, particularly casein-sIgE, which should be considered when interpreting results across platforms.

Despite advances in laboratory diagnostics, OFCs remain the reference standard for confirming food allergy and assessing the development of tolerance. Available diagnostic tests may support patient selection and risk stratification before challenge procedures but cannot replace OFCs in clinical practice.

Given the retrospective design, potential spectrum bias, and relatively small sample size, the proposed diagnostic thresholds should be regarded as preliminary and require validation in larger, prospective, independent cohorts before they can be considered for routine clinical decision-making. Further studies are also needed to clarify the role of component-resolved diagnostics across different clinical phenotypes of food allergy and across diagnostic platforms.

## Figures and Tables

**Table 1:** Doses of milk and egg during oral food challenges.

Dose Number	Boiled Egg		Raw Milk		Baked Egg (Muffins)		Baked Milk (Muffins)	
	Food mg	Protein mg	Food mL	Protein mg	Food amount ^1^	Protein mg	Food amount ^1^	Protein mg
1	100	13	1.2	40	¼	800	¼	320
2	500	65	3.1	100	½	1600	½	640
3	2500	325	12.5	400	¾	2400	¾	960
4	8000	1040	25	800	1 and ½	4800	1 and ½	1920
5	25,000	3250	37.5	1200				
6	25,000 ^2^	3250	50 or 112 ^3^	1600 or 3600 ^3^				
7			125 ^4^	4000				

^1^ Portion of a muffin. ^2^ Only in children older than 4 yo. ^3^ In children above 8 yo dose 50 mL (1600 mg) and in children under 8 yo dose 112 mL (3600 mg). ^4^ Only in children under 8 yo.

**Table 2:** Basic characteristics of children undergoing OFC to baked milk.

Characteristic	Total Group (*n* = 42) ^1^	OFC+ (*n* = 13) ^1^	OFC− (*n* = 29) ^1^	*p*-Value ^2^
Age, median (IQR)	6.1 (3.1–9.5)	5.4 (3.1–6.6)	6.7 (3.5–10.3)	0.301
Gender				0.163
Male	27 (64.3%)	6 (46.2%)	21 (72.4%)	
Female	15 (35.7%)	7 (53.8%)	8 (27.6%)	
Atopic dermatitis, *n* (%)	27 (64.3%)	9 (69.2%)	18 (62.1%)	0.739
Allergic rhinitis, *n* (%)	23 (54.8%)	4 (30.8%)	19 (65.5%)	**0.049**
Asthma, *n* (%)	16 (38.1%)	5 (38.5%)	11 (37.9%)	>0.999
Other food allergies, *n* (%)	35 (83.3%)	10 (76.9%)	25 (86.2%)	0.657
Past allergic reaction to milk, *n* (%)	26 (61.9%)	10 (76.9%)	16 (55.2%)	0.303
Allergic reaction within 1 year, *n* (%)	5 (11.9%)	2 (15.4%)	3 (10.3%)	0.637
Past anaphylaxis, *n* (%)	20 (47.6%)	7 (53.8%)	13 (44.8%)	0.741

^1^ Median (IQR) for age; *n* (%) for categorical variables. ^2^ Comparison between OFC+ and OFC−. Mann–Whitney U test for age; Fisher’s exact test for categorical. Bold formatting was used to highlight statistically significant differences between the evaluated parameters.

**Table 3:** Comparison of SPT and sIgE results in children with positive and negative OFC outcome to baked milk.

Parameter ^1^	OFC+ (*n* = 13) ^2^	OFC− (*n* = 29) ^2^	*p*-Value
SPT raw milk	10.00 (9.00–17.00)	10.00 (7.00–12.00)	0.142
SPT milk extract	7.00 (5.00–12.00)	6.00 (5.00–8.00)	0.197
sIgE milk extract ImmunoCAP	20.55 (10.48–40.72)	5.60 (3.03–12.30)	**0.007**
sIgE α-lactalbumin ImmunoCAP	10.40 (2.06–31.60)	3.44 (0.20–8.50)	0.142
sIgE β-lactoglobulin ImmunoCAP	3.28 (2.00–17.50)	0.30 (0.10–1.90)	**0.015**
sIgE casein ImmunoCAP	13.64 (8.56–22.83)	3.04 (0.67–7.57)	**0.001**
sIgE milk extract ALEX2	8.63 (5.33–11.61)	2.93 (1.16–6.61)	**0.010**
sIgE α-lactalbumin ALEX2	4.06 (0.68–17.53)	1.69 (0.10–6.13)	0.358
sIgE β-lactoglobulin ALEX2	3.86 (1.61–6.12)	0.47 (0.10–2.48)	**0.026**
sIgE casein ALEX2	7.00 (5.85–9.85)	3.14 (2.09–6.14)	**0.024**

^1^ Sizes of SPT are given as mm, values of sIgE are given as kUA/L. ^2^ Median (IQR). Bold formatting was used to highlight statistically significant differences between the evaluated parameters.

**Table 4:** Predictive performance of single parameters for positive OFC to baked milk.

Predictor	AUC	Cut-Off *	Se	Sp	PPV	NPV	LR+	PPV ≥ 95% Cut-Off	NPV ≥ 90% Cut-Off
sIgE casein ImmunoCAP	0.842 (0.709–0.975)	5.27 (5.27–19.90)	100% (69–100)	62% (41–81)	53% (29–76)	100% (78–100)	2.67	22.14 (14.80–41.20)	7.44 (4.17–22.14)
sIgE milk extract ImmunoCAP	0.805 (0.651–0.959)	6.18 (6.18–25.20)	100% (69–100)	57% (34–78)	53% (29–76)	100% (74–100)	2.33	-	7.59 (5.11–23.70)
sIgE milk extract ALEX2	0.793 (0.635–0.951)	3.12 (3.12–10.45)	100% (69–100)	52% (30–74)	50% (27–73)	100% (72–100)	2.10	-	4.26 (2.67–10.36)
sIgE β-lactoglobulin ImmunoCAP	0.783 (0.615–0.950)	2.00 (0.22–17.50)	78% (40–97)	74% (52–90)	54% (25–81)	89% (67–99)	2.98	53.70 (10.86–54.25)	0.44 (0.17–34.83)
sIgE casein ALEX2	0.757 (0.576–0.938)	5.56 (2.31–7.06)	80% (44–97)	71% (48–89)	57% (29–82)	88% (64–99)	2.80	-	3.15 (1.23–6.76)
sIgE β-lactoglobulin ALEX2	0.752 (0.561–0.944)	1.19 (1.19–6.55)	90% (55–100)	62% (38–82)	53% (28–77)	93% (66–100)	2.36	20.92 (4.46–22.96)	0.94 (0.81–4.47)

AUC: area under ROC curve (95% CI, DeLong). Youden cut-off: threshold maximizing Youden index, with bootstrap 95% CI (2000 runs). PPV ≥ 95% cut-off: lowest threshold achieving positive predictive value ≥ 95% (rule-in), with bootstrap 95% CI. NPV ≥ 90% cut-off: highest threshold achieving negative predictive value ≥ 90% (rule-out), with bootstrap 95% CI. ‘-’ indicates target PPV or NPV was not achievable in this dataset. PPV and NPV are prevalence-dependent and should not be extrapolated to populations with substantially different disease prevalence. Se: sensitivity; Sp: specificity; LR+: positive likelihood ratio. All proportions with Wilson 95% CI. * proposed thresholds should be regarded as exploratory rather than definitive clinical decision points.

**Table 5:** Basic characteristics of children undergoing OFC to raw milk.

Characteristic	Total Group (*n* = 43) ^1^	OFC+ (*n* = 18) ^1^	OFC− (*n* = 25) ^1^	*p*-Value ^2^
Age, median (IQR)	6.0 (3.8–8.1)	5.9 (3.5–10.3)	6.1 (3.8–7.5)	0.694
Gender				0.731
Male	31 (72.1%)	14 (77.8%)	17 (68.0%)	
Female	12 (27.9%)	4 (22.2%)	8 (32.0%)	
Atopic dermatitis, *n* (%)	21 (48.8%)	10 (55.6%)	11 (44.0%)	0.543
Allergic rhinitis, *n* (%)	19 (45.2%)	9 (52.9%)	10 (40.0%)	0.531
Asthma, *n* (%)	19 (44.2%)	9 (50.0%)	10 (40.0%)	0.550
Other food allergies (different than milk), *n* (%)	31 (72.1%)	14 (77.8%)	17 (68.0%)	0.731
Tolerance to baked milk, *n* (%)	38 (88.4%)	16 (88.8%)	22 (88.0%)	>0.999
Allergic reaction within one year, *n* (%)	4 (9.5%)	0 (0.0%)	4 (16.7%)	0.122
Past allergic reaction to raw milk, *n* (%)	22 (51.2%)	12 (66.7%)	10 (40.0%)	0.124
Anaphylactic reaction, *n* (%)	17 (40.5%)	6 (33.3%)	11 (44.0%)	0.539

^1^ Median (IQR) for age; *n* (%) for categorical variables. ^2^ Comparison between OFC+ and OFC−. Mann–Whitney U test for age; Fisher’s exact test for categorical.

**Table 6:** Comparison of SPT and sIgE results in children with positive and negative OFC outcome to raw milk.

Parameter ^1^	OFC+ (*n* = 18) ^2^	OFC− (*n* = 25) ^2^	*p*-Value
SPT raw milk	8.00 (5.00–10.00)	5.00 (4.00–7.00)	**0.0355**
SPT milk extract	4.00 (3.00–5.75)	4.00 (0.00–5.00)	0.533
sIgE milk extract ImmunoCAP	5.85 (3.40–7.32)	1.83 (0.58–5.12)	**0.0284**
sIgE α-lactalbumin ImmunoCAP	3.48 (0.37–5.81)	0.14 (0.04–0.80)	**0.0206**
sIgE β-lactoglobulin ImmunoCAP	1.24 (0.14–3.54)	0.23 (0.13–1.34)	0.226
sIgE casein ImmunoCAP	2.29 (0.63–4.41)	0.80 (0.35–2.54)	0.107
sIgE milk extract ALEX2	1.00 (0.66–2.88)	0.40 (0.10–1.07)	0.132
sIgE α-lactalbumin ALEX2	1.20 (0.10–4.36)	0.10 (0.10–0.66)	0.114
sIgE β-lactoglobulin ALEX2	1.41 (0.43–2.40)	0.22 (0.10–1.35)	**0.027**
sIgE casein ALEX2	0.51 (0.21–3.72)	0.35 (0.10–0.94)	0.187

^1^ Sizes of SPT are given as mm, values of sIgE are given as kUA/L. ^2^ Median (IQR). Bold formatting was used to highlight statistically significant differences between the evaluated parameters.

**Table 7:** Predictive performance of single parameters for positive OFC to raw milk.

Predictor	AUC	Cut-Off *	Se	Sp	PPV	NPV	LR+	PPV ≥ 95% Cut-Off	NPV ≥ 90% Cut-Off
sIgE β -lactoglobulin ALEX2	0.733 (0.548–0.918)	0.28 (0.28–2.40)	85% (55–98)	58% (33–80)	58% (33–80)	85% (55–98)	2.01	5.49 (1.75–7.16)	-
sIgE α-lactalbumin ImmunoCAP	0.726 (0.550–0.903)	2.83 (0.30–4.51)	56% (30–80)	90% (70–99)	82% (48–98)	73% (52–88)	5.91	-	0.03 (0.02–2.05)
sIgE milk extract IC	0.714 (0.541–0.888)	3.03 (1.06–6.10)	81% (54–96)	71% (48–89)	68% (43–87)	83% (59–96)	2.84	24.55 (6.67–24.55)	0.24 (0.20–3.26)
SPT raw milk	0.692 (0.527–0.857)	10.00 (5.00–12.00)	47% (23–72)	84% (64–95)	67% (35–90)	70% (51–85)	2.94	13.50 (11.00–15.00)	-

AUC: area under ROC curve (95% CI, DeLong). Youden cut-off: threshold maximizing Youden index, with bootstrap 95% CI (2000 runs). PPV ≥ 95% cut-off: lowest threshold achieving positive predictive value ≥ 95% (rule-in), with bootstrap 95% CI. NPV ≥ 90% cut-off: highest threshold achieving negative predictive value ≥ 90% (rule-out), with bootstrap 95% CI. ‘-’ indicates target PPV or NPV was not achievable in this dataset. PPV and NPV are prevalence-dependent and should not be extrapolated to populations with substantially different disease prevalence. Se: sensitivity; Sp: specificity; LR+: positive likelihood ratio. All proportions with Wilson 95% CI. * proposed thresholds should be regarded as exploratory rather than definitive clinical decision points.

**Table 8:** Basic characteristics of children undergoing OFC to boiled egg.

Characteristic	Total Group (*n* = 54) ^1^	OFC+ (*n* = 18) ^1^	OFC− (*n* = 36) ^1^	*p*-Value ^2^
Age, median (IQR)	5.8 (3.3–8.9)	5.8 (4.0–8.9)	5.4 (3.3–9.5)	0.797
Gender				0.339
Male	40 (74.1%)	15 (83.3%)	25 (69.4%)	
Female	14 (25.9%)	3 (16.7%)	11 (30.6%)	
Atopic dermatitis, *n* (%)	35 (64.8%)	11 (61.1%)	24 (66.7%)	0.766
Allergic rhinitis, *n* (%)	24 (44.4%)	9 (50.0%)	15 (41.7%)	0.577
Asthma, *n* (%)	18 (33.3%)	7 (38.9%)	11 (30.6%)	0.556
Other food allergies, *n* (%)	45 (83.3%)	15 (83.3%)	30 (83.3%)	>0.999
Tolerance to baked egg, *n* (%)	46 (85.2%)	14 (77.8%)	32 (88.9%)	0.418
Past allergic reaction to egg, *n* (%)	28 (51.9%)	9 (50.0%)	19 (52.8%)	>0.999
Allergic reaction within one year, *n* (%)	0 (0.0%)	0 (0.0%)	0 (0.0%)	>0.999
Past anaphylaxis, *n* (%)	17 (31.5%)	6 (33.3%)	11 (30.6%)	>0.999

^1^ Median (IQR) for age; *n* (%) for categorical variables. ^2^ Comparison between OFC+ and OFC−. Mann–Whitney U test for age; Fisher’s exact test for categorical.

**Table 9:** Comparison of SPT and sIgE results in children with positive and negative OFC outcome to boiled egg.

Parameter ^1^	OFC+ (*n* = 18) ^2^	OFC− (*n* = 36) ^2^	*p*-Value
SPT egg white extract	5.00 (4.00–6.00)	5.00 (3.00–7.00)	0.535
SPT egg yolk extract	4.50 (3.00–6.00)	3.00 (0.00–5.00)	**0.042**
SPT egg white raw	10.00 (9.00–15.00)	8.00 (6.00–10.00)	**0.010**
SPT egg yolk raw	5.00 (3.50–7.50)	2.00 (0.00–5.00)	**0.010**
sIgE egg extract ImmunoCAP	3.26 (1.43–5.98)	1.07 (0.66–2.46)	0.121
sIgE egg white ImmunoCAP	2.34 (1.12–6.51)	0.93 (0.43–1.88)	0.078
sIgE egg yolk ImmunoCAP	1.08 (0.49–2.56)	0.24 (0.17–0.69)	0.072
sIgE egg white extract ALEX2	4.72 (2.17–8.06)	0.49 (0.27–3.12)	**0.012**
sIgE egg yolk extract ALEX2	0.26 (0.16–0.82)	0.11 (0.10–0.23)	**0.023**
sIgE ovomucoid ALEX2	1.35 (0.70–5.12)	0.21 (0.10–1.14)	**0.022**
sIgE ovalbumin ALEX2	1.53 (0.77–5.10)	0.45 (0.10–2.23)	**0.035**
sIgE ovotransferrin ALEX2	0.48 (0.10–1.59)	0.10 (0.10–0.43)	0.074

^1^ Sizes of SPT are given as mm, values of sIgE are given as kUA/L. ^2^ Median (IQR). Bold formatting was used to highlight statistically significant differences between the evaluated parameters.

**Table 10:** Predictive performance of single parameters for positive OFC to boiled egg.

Predictor	AUC	Cut-Off *	Se	Sp	PPV	NPV	LR+	PPV ≥ 95% Cut-Off	NPV ≥ 90% Cut-Off
SPT egg yolk raw	0.738 (0.586–0.890)	4.00 (2.00–7.00)	73% (45–92)	69% (49–85)	55% (32–77)	83% (63–95)	2.36	15.50 (6.50–15.50)	1.00 (1.00–4.00)
sIgE egg white extract ALEX2	0.735 (0.576–0.894)	3.36 (0.25–7.82)	69% (41–89)	76% (55–91)	65% (38–86)	79% (58–93)	2.86	15.82 (6.83–15.82)	0.23 (0.17–3.59)
SPT egg white raw	0.725 (0.580–0.870)	8.00 (7.00–15.00)	88% (64–99)	48% (31–66)	47% (29–65)	89% (65–99)	1.71	-	6.50 (2.00–9.50)
sIgE ovomucoid ALEX2	0.713 (0.543–0.882)	0.46 (0.46–4.87)	81% (54–96)	60% (39–79)	57% (34–77)	83% (59–96)	2.03	15.72 (2.96–19.12)	-
sIgE egg yolk extract ALEX2	0.709 (0.544–0.873)	0.16 (0.16–1.16)	81% (54–96)	64% (43–82)	59% (36–79)	84% (60–97)	2.26	-	-
sIgE ovalbumin ALEX2	0.698 (0.532–0.863)	0.45 (0.16–5.50)	88% (62–98)	48% (28–69)	52% (32–71)	86% (57–98)	1.68	15.88 (3.26–15.88)	-
SPT egg yolk extract	0.681 (0.517–0.846)	4.00 (1.00–7.00)	62% (35–85)	70% (51–85)	53% (29–76)	78% (58–91)	2.08	6.50 (6.50–15.50)	-

AUC: area under ROC curve (95% CI, DeLong). Youden cut-off: threshold maximizing Youden index, with bootstrap 95% CI (2000 runs). PPV ≥ 95% cut-off: lowest threshold achieving positive predictive value ≥ 95% (rule-in), with bootstrap 95% CI. NPV ≥ 90% cut-off: highest threshold achieving negative predictive value ≥ 90% (rule-out), with bootstrap 95% CI. ‘-’ indicates target PPV or NPV was not achievable in this dataset. PPV and NPV are prevalence-dependent and should not be extrapolated to populations with substantially different disease prevalence. Se: sensitivity; Sp: specificity; LR+: positive likelihood ratio. All proportions with Wilson 95% CI. * proposed thresholds should be regarded as exploratory rather than definitive clinical decision points.

**Table 11:** Basic characteristics of children undergoing OFC to baked egg.

Characteristic	Total Group (*n* = 54) ^1^	OFC+ (*n* = 15) ^1^	OFC− (*n* = 39) ^1^	*p*-Value ^2^
Age, median (IQR)	5.3 (3.4–7.8)	5.4 (3.3–8.3)	5.3 (3.4–7.7)	0.954
Gender				>0.999
Male	34 (63.0%)	10 (66.7%)	24 (61.5%)	
Female	20 (37.0%)	5 (33.3%)	15 (38.5%)	
Atopic dermatitis, *n* (%)	35 (64.8%)	13 (86.7%)	22 (56.4%)	0.056
Allergic rhinitis, *n* (%)	33 (61.1%)	13 (86.7%)	20 (51.3%)	**0.028**
Asthma, *n* (%)	22 (40.7%)	9 (60.0%)	13 (33.3%)	0.121
Other food allergies, *n* (%)	47 (87.0%)	15 (100.0%)	32 (82.1%)	0.171
Past allergic reaction to egg, *n* (%)	35 (64.8%)	11 (73.3%)	24 (61.5%)	0.532
Allergic reaction within one year, *n* (%)	2 (3.7%)	1 (6.7%)	1 (2.6%)	0.482
Past anaphylaxis, *n* (%)	16 (29.6%)	9 (60.0%)	7 (17.9%)	0.006

^1^ Median (IQR) for age; *n* (%) for categorical variables. ^2^ Comparison between OFC+ and OFC−. Mann–Whitney U test for age; Fisher’s exact test for categorical. Bold formatting was used to highlight statistically significant differences between the evaluated parameters.

**Table 12:** Comparison of SPT and sIgE results in children with positive and negative OFC outcome to baked egg.

Parameter ^1^	OFC+ (*n* = 15) ^2^	OFC− (*n* = 39) ^2^	*p*-Value
SPT egg white extract	11.00 (8.00–15.00)	7.00 (5.00–9.00)	**0.005**
SPT egg yolk extract	7.00 (4.00–9.00)	5.00 (3.50–6.00)	0.267
SPT egg white raw	13.00 (12.75–16.25)	12.00 (10.00–15.00)	0.196
SPT egg yolk raw	7.50 (6.00–11.25)	5.00 (3.75–9.00)	0.059
sIgE egg extract ImmunoCAP	11.85 (7.21–17.58)	7.42 (2.66–22.93)	0.461
sIgE egg white ImmunoCAP	10.25 (7.16–15.33)	5.14 (1.96–18.95)	0.369
sIgE egg yolk ImmunoCAP	4.53 (2.75–6.74)	2.15 (0.66–4.75)	0.096
sIgE egg white extract ALEX2	13.92 (9.48–21.68)	5.64 (2.75–10.16)	**0.028**
sIgE egg yolk extract ALEX2	1.26 (0.67–1.88)	0.57 (0.10–1.25)	**0.042**
sIgE ovomucoid ALEX2	7.58 (5.05–23.12)	2.84 (0.33–6.12)	0.078
sIgE ovalbumin ALEX2	10.09 (9.12–26.33)	3.98 (0.91–5.87)	**0.004**
sIgE ovotransferrin ALEX2	4.57 (1.03–6.50)	0.87 (0.14–2.19)	**0.026**

^1^ Sizes of SPT are given as mm, values of sIgE are given as kUA/L. ^2^ Median (IQR). Bold formatting was used to highlight statistically significant differences between the evaluated parameters.

**Table 13:** Predictive performance of single parameters for positive OFC to baked egg.

Predictor	AUC	Cut-Off *	Se	Sp	PPV	NPV	LR+	PPV ≥ 95% Cut-Off	NPV ≥ 90% Cut-Off
sIgE ovalbumin ALEX2	0.853 (0.718–0.988)	8.74 (4.96–21.71)	86% (42–100)	84% (67–95)	55% (23–83)	96% (82–100)	5.49	-	9.25 (4.75–32.08)
sIgE ovotransferrin ALEX2	0.772 (0.568–0.977)	4.57 (0.55–7.47)	57% (18–90)	97% (84–100)	80% (28–99)	91% (76–98)	18.29	-	4.50 (0.44–5.95)
SPT egg white extract	0.762 (0.602–0.922)	10.00 (6.00–14.00)	69% (39–91)	81% (65–92)	56% (30–80)	88% (73–97)	3.66	15.50 (15.50–17.50)	5.50 (4.50–13.00)
sIgE egg white extract ALEX2	0.754 (0.558–0.950)	9.29 (9.29–22.05)	88% (47–100)	65% (45–81)	39% (17–64)	95% (76–100)	2.47	26.27 (20.98–26.27)	9.01 (2.08–26.27)
sIgE egg yolk extract ALEX2	0.736 (0.555–0.916)	0.39 (0.39–1.82)	100% (63–100)	45% (27–64)	32% (15–54)	100% (77–100)	1.82	-	0.61 (0.29–2.38)
SPT egg yolk raw	0.719 (0.553–0.884)	6.00 (5.00–11.00)	88% (47–100)	59% (41–76)	35% (15–59)	95% (75–100)	2.15	23.00 (19.50–23.00)	5.50 (4.50–23.00)

AUC: area under ROC curve (95% CI, DeLong). Youden cut-off: threshold maximizing Youden index, with bootstrap 95% CI (2000 runs). PPV ≥ 95% cut-off: lowest threshold achieving positive predictive value ≥ 95% (rule-in), with bootstrap 95% CI. NPV ≥ 90% cut-off: highest threshold achieving negative predictive value ≥90% (rule-out), with bootstrap 95% CI. ‘-’ indicates target PPV or NPV was not achievable in this dataset. PPV and NPV are prevalence-dependent and should not be extrapolated to populations with substantially different disease prevalence. Se: sensitivity; Sp: specificity; LR+: positive likelihood ratio. All proportions with Wilson 95% CI. * proposed thresholds should be regarded as exploratory rather than definitive clinical decision points.

## Data Availability

Data are not openly available due to ethical/privacy restrictions as permission for data sharing was not provided by the participants or the Ethical Committee.

## Supplementary Materials

The following supporting information can be downloaded at: https://www.mdpi.com/article/10.3390/nu18132157/s1, Table S1: Comparison of total IgE levels and sIgE-to-total IgE ratios, both determined using the ALEX2 platform, in children with positive and negative OFC outcomes to baked milk; Table S2: Two-predictor logistic regression models for predicting positive OFC to baked milk; Table S3: Comparison of total IgE levels and sIgE-to-total IgE ratios, both determined using the ALEX2 platform, in children with positive and negative OFC outcomes to raw milk; Table S4: Two-predictor logistic regression models for predicting positive OFC to raw milk; Table S5: Quantitative and qualitative agreement between ALEX2 and ImmunoCAP for results of milk components-sIgE (combined raw milk + baked milk cohorts, *n* = 85); Table S6: Predictive performance comparison (paired): ALEX2 vs. ImmunoCAP (baked milk cohort); Table S7: Predictive performance comparison (paired): ALEX2 vs. ImmunoCAP (raw milk cohort); Table S8: Comparison of total IgE levels and sIgE-to-total IgE ratios, both determined using the ALEX2 platform, in children with positive and negative OFC outcomes to boiled egg; Table S9: Two-predictor logistic regression models for predicting positive OFC to boiled egg; Table S10: Comparison of total IgE levels and sIgE-to-total IgE ratios, both determined using the ALEX2 platform, in children with positive and negative OFC outcomes to baked egg; Table S11: Two-predictor logistic regression models for predicting positive OFC to baked egg; Table S12: Quantitative and qualitative agreement between results of ALEX2 and ImmunoCAP for results of egg extracts-sIgE (combined boiled egg + baked egg cohorts, *n* = 108); Table S13: Predictive performance comparison (paired): ALEX2 vs. ImmunoCAP for egg extracts (boiled egg cohort); Table S14: Predictive performance comparison (paired): ALEX2 vs. ImmunoCAP for egg extracts (baked egg cohort).

## Abbreviations

The following abbreviations are used in this manuscript:

CM	Cow’s Milk
HE	Hen’s Egg
BAT	Basophil Activation Test
sIgE	Specific Immunoglobulin E
OFC	Oral Food Challenge
SPT	Skin Prick Test

## References

[1] Spolidoro G.C.I., Ali M.M., Amera Y.T., Nyassi S., Lisik D., Ioannidou A., Rovner G., Khaleva E., Venter C., van Ree R. (2023). Prevalence estimates of eight big food allergies in Europe: Updated systematic review and meta-analysis. Allergy.

[2] Schoemaker A.A., Sprikkelman A.B., Grimshaw K.E., Roberts G., Grabenhenrich L., Rosenfeld L., Siegert S., Dubakiene R., Rudzeviciene O., Reche M. (2015). Incidence and natural history of challenge-proven cow’s milk allergy in European children--EuroPrevall birth cohort. Allergy.

[3] Xepapadaki P., Fiocchi A., Grabenhenrich L., Roberts G., Grimshaw K.E., Fiandor A., Larco J.I., Sigurdardottir S., Clausen M., Papadopoulos N.G. (2016). Incidence and natural history of hen’s egg allergy in the first 2 years of life-the EuroPrevall birth cohort study. Allergy.

[4] Nwaru B.I., Hickstein L., Panesar S.S., Roberts G., Muraro A., Sheikh A. (2014). Prevalence of common food allergies in Europe: A systematic review and meta-analysis. Allergy.

[5] Santos A.F., Riggioni C., Agache I., Akdis C.A., Akdis M., Alvarez-Perea A., Alvaro-Lozano M., Ballmer-Weber B., Barni S., Beyer K. (2023). EAACI guidelines on the diagnosis of IgE-mediated food allergy. Allergy.

[6] Hansen M.M., Nissen S.P., Halken S., Høst A. (2021). The natural course of cow’s milk allergy and the development of atopic diseases into adulthood. Pediatr. Allergy Immunol..

[7] Peters R.L., Koplin J.J., Gurrin L.C., Dharmage S.C., Wake M., Ponsonby A.L., Tang M.L.K., Lowe A.J., Matheson M., Dwyer T. (2017). The prevalence of food allergy and other allergic diseases in early childhood in a population-based study: HealthNuts age 4-year follow-up. J. Allergy Clin. Immunol..

[8] Gonzalez P.M., Cassin A.M., Durban R., Upton J.E.M. (2025). Effects of Food Processing on Allergenicity. Curr. Allergy Asthma Rep..

[9] Leonard S.A., Caubet J.C., Kim J.S., Groetch M., Nowak-Węgrzyn A. (2015). Baked milk- and egg-containing diet in the management of milk and egg allergy. J. Allergy Clin. Immunol. Pract..

[10] Esmaeilzadeh H., Alyasin S., Haghighat M., Nabavizadeh H., Esmaeilzadeh E., Mosavat F. (2018). The effect of baked milk on accelerating unheated cow’s milk tolerance: A control randomized clinical trial. Pediatr. Allergy Immunol..

[11] Netting M., Gold M., Quinn P., El-Merhibi A., Penttila I., Makrides M. (2017). Randomised controlled trial of a baked egg intervention in young children allergic to raw egg but not baked egg. World Allergy Organ. J..

[12] Sampson H.A., Arasi S., Bahnson H.T., Ballmer-Weber B., Beyer K., Bindslev-Jensen C., Bird J.A., Blumchen K., Davis C., Ebisawa M. (2024). AAAAI-EAACI PRACTALL: Standardizing oral food challenges-2024 Update. Pediatr. Allergy Immunol..

[13] Patel N., Shreffler W.G., Custovic A., Santos A.F. (2023). Will Oral Food Challenges Still Be Part of Allergy Care in 10 Years’ Time?. J. Allergy Clin. Immunol. Pract..

[14] Riggioni C., Ricci C., Moya B., Wong D., van Goor E., Bartha I., Buyuktiryaki B., Giovannini M., Jayasinghe S., Jaumdally H. (2024). Systematic review and meta-analyses on the accuracy of diagnostic tests for IgE-mediated food allergy. Allergy.

[15] Bartha I., Boyd H., Foong R.X., Krawiec M., Marques-Mejias A., Marshall H.F., Radulovic S., Harrison F., Antoneria G., Jama Z. (2025). The Basophil Activation Test Is the Most Accurate Test in Predicting Allergic Reactions to Baked and Fresh Cow’s Milk During Oral Food Challenges. Allergy.

[16] Krawiec M., Radulovic S., Foong R.X., Marques-Mejias A., Bartha I., Kwok M., Jama Z., Harrison F., Ricci C., Lack G. (2023). Diagnostic utility of allergy tests to predict baked egg and lightly cooked egg allergies compared to double-blind placebo-controlled food challenges. Allergy.

[17] Domínguez O., Riggioni C., Poyatos E., Jiménez-Feijoo R.M., Piquer M., Machinena A., Folqué M., Ortiz de Landazuri I., Torradeflot M., Lozano J. (2026). Biomarkers of Tolerance to Baked Milk in Cow’s Milk-Allergic Children at High Risk of Anaphylaxis. J. Investig. Allergol. Clin. Immunol..

[18] Cogurlu M.T., Uluc N.N., Ozanli I., Ozkan Y.E., Iskender N., Balci S., Simsek I.E., Aydogan M. (2025). The utility of casein skin prick test and IgE values in predicting anaphylaxis and reactivity to baked milk. Ann. Allergy Asthma Immunol..

[19] Caubet J.C., Nowak-Węgrzyn A., Moshier E., Godbold J., Wang J., Sampson H.A. (2013). Utility of casein-specific IgE levels in predicting reactivity to baked milk. J. Allergy Clin. Immunol..

[20] Vilar L.K., Araújo F.A., Santos T.P., Menezes T.T., Cheik M.F., Segundo G.R.S. (2021). Baked Tolerance in Cow’s Milk Allergy: Quite Frequent, Hard to Predict!. Int. Arch. Allergy Immunol..

[21] Coelho P.S., Santos G.M.D., Sangalho I., Rosa S., Pinto P.L. (2024). Role of serum-specific immunoglobulin E in egg allergy: A comprehensive study of Portuguese pediatric patients. Allergol. Immunopathol..

[22] Bartnikas L.M., Sheehan W.J., Larabee K.S., Petty C., Schneider L.C., Phipatanakul W. (2013). Ovomucoid is not superior to egg white testing in predicting tolerance to baked egg. J. Allergy Clin. Immunol. Pract..

[23] Wang J., Godbold J.H., Sampson H.A. (2008). Correlation of serum allergy (IgE) tests performed by different assay systems. J. Allergy Clin. Immunol..

[24] Majsiak E., Choina M., Miśkiewicz K., Pukalyak S., Smolińska S., Kurzawa R. (2025). A Comparison of asIgE Levels Measured with ALEX and ImmunoCAP ISAC in Polish Children with Food Allergies. Int. J. Mol. Sci..

[25] Čelakovská J., Cermakova E., Vankova R., Boudková P., Andrys C., Krejsek J. (2023). Sensitivity, specificity and positive predictive value of ALEX2 multiplex examination in patients suffering from atopic dermatitis and reaction to tomatoes. Food Agric. Immunol..

[26] Altunbas M.Y., Gungoren E.Y., Can S., Amirov R., Ozturk N., Bozkurt S., Bilgic Eltan S., Baris S., Ozen A., Karakoc-Aydiner E. (2026). Early markers of baked milk and egg tolerance in young children with IgE-mediated immediate reactions. Eur. Ann. Allergy Clin. Immunol..

[27] Cogurlu M.T., Simsek I.E., Aydogan M., Uncuoglu A., Acar H.C. (2022). Prospective evaluation of tolerance to unheated milk-boiled egg after baked milk-egg tolerance under 2 years. Ann. Allergy Asthma Immunol..

[28] Sampson H.A., Gerth van Wijk R., Bindslev-Jensen C., Sicherer S., Teuber S.S., Burks A.W., Dubois A.E., Beyer K., Eigenmann P.A., Spergel J.M. (2012). Standardizing double-blind, placebo-controlled oral food challenges: American Academy of Allergy, Asthma & Immunology-European Academy of Allergy and Clinical Immunology PRACTALL consensus report. J. Allergy Clin. Immunol..

[29] Sicherer S.H., Wood R.A., Vickery B.P., Perry T.T., Jones S.M., Leung D.Y., Blackwell B., Dawson P., Burks A.W., Lindblad R. (2016). Impact of Allergic Reactions on Food-Specific IgE Concentrations and Skin Test Results. J. Allergy Clin. Immunol. Pract..

[30] Blazowski L., Majak P., Kurzawa R., Kuna P., Jerzynska J. (2021). A severity grading system of food-induced acute allergic reactions to avoid the delay of epinephrine administration. Ann. Allergy Asthma Immunol..

[31] Turner P.J., Ansotegui I.J., Campbell D.E., Cardona V., Carr S., Custovic A., Durham S., Ebisawa M., Geller M., Gonzalez-Estrada A. (2024). Updated grading system for systemic allergic reactions: Joint Statement of the World Allergy Organization Anaphylaxis Committee and Allergen Immunotherapy Committee. World Allergy Organ. J..

[32] DeLong E.R., DeLong D.M., Clarke-Pearson D.L. (1988). Comparing the areas under two or more correlated receiver operating characteristic curves: A nonparametric approach. Biometrics.

[33] Ruopp M.D., Perkins N.J., Whitcomb B.W., Schisterman E.F. (2008). Youden Index and optimal cut-point estimated from observations affected by a lower limit of detection. Biom. J..

[34] Bartnikas L.M., Sheehan W.J., Hoffman E.B., Permaul P., Dioun A.F., Friedlander J., Baxi S.N., Schneider L.C., Phipatanakul W. (2012). Predicting food challenge outcomes for baked milk: Role of specific IgE and skin prick testing. Ann. Allergy Asthma Immunol..

[35] Esty B., Maciag M.C., Bartnikas L.M., Petty C.R., MacGinnitie A.J., Sheehan W.J., Phipatanakul W. (2021). Predicting outcomes of baked egg and baked milk oral food challenges by using a ratio of food-specific IgE to total IgE. J. Allergy Clin. Immunol. Pract..

[36] Kiykim A., Karakoc-Aydiner E., Gunes E., Nain E., Ogulur I., Yazici D., Aktac S., Bicer A.H., Sackesen C., Baris S. (2019). Evaluation of a Standardized Bakery Product (SUTMEK) as a Potential Tool for Baked-Milk Tolerance and Immunotherapy Research Studies. Int. Arch. Allergy Immunol..

[37] Kwan A., Asper M., Lavi S., Lavine E., Hummel D., Upton J.E. (2016). Prospective evaluation of testing with baked milk to predict safe ingestion of baked milk in unheated milk-allergic children. Allergy Asthma Clin. Immunol..

[38] Sirin Kose S., Asilsoy S., Uzuner N., Karaman O., Anal O. (2019). Outcomes of Baked Milk and Egg Challenge in Cow’s Milk and Hen’s Egg Allergy: Can Tolerance Be Predicted with Allergen-Specific IgE and Prick-to-Prick Test?. Int. Arch. Allergy Immunol..

[39] Nowak-Wegrzyn A., Bloom K.A., Sicherer S.H., Shreffler W.G., Noone S., Wanich N., Sampson H.A. (2008). Tolerance to extensively heated milk in children with cow’s milk allergy. J. Allergy Clin. Immunol..

[40] Nieminen O., Palosuo K., Mäkelä M.J. (2025). Casein sIgE as the most accurate predictor for heated milk tolerance in Finnish children. Pediatr. Allergy Immunol..

[41] Čurlej J., Zajác P., Čapla J., Golian J., Benešová L., Partika A., Fehér A., Jakabová S. (2022). The Effect of Heat Treatment on Cow’s Milk Protein Profiles. Foods.

[42] Bavaro S.L., De Angelis E., Barni S., Pilolli R., Mori F., Novembre E.M., Monaci L. (2019). Modulation of Milk Allergenicity by Baking Milk in Foods: A Proteomic Investigation. Nutrients.

[43] Kim J.S., Nowak-Węgrzyn A., Sicherer S.H., Noone S., Moshier E.L., Sampson H.A. (2011). Dietary baked milk accelerates the resolution of cow’s milk allergy in children. J. Allergy Clin. Immunol..

[44] Demir Şahin F., Kapçay O., Kılıç M., Şahin Sindi H. (2026). Diagnostic Utility of Skin Prick Test Ratios and Specific IgE in Predicting Egg White Allergy: Reducing the Need for Oral Food Challenges in Children. J. Clin. Med..

[45] Alessandri C., Zennaro D., Scala E., Ferrara R., Bernardi M.L., Santoro M., Palazzo P., Mari A. (2012). Ovomucoid (Gal d 1) specific IgE detected by microarray system predict tolerability to boiled hen’s egg and an increased risk to progress to multiple environmental allergen sensitisation. Clin. Exp. Allergy.

[46] Calvani M., Arasi S., Bianchi A., Caimmi D., Cuomo B., Dondi A., Indirli G.C., La Grutta S., Panetta V., Verga M.C. (2015). Is it possible to make a diagnosis of raw, heated, and baked egg allergy in children using cutoffs? A systematic review. Pediatr. Allergy Immunol..

[47] Ando H., Movérare R., Kondo Y., Tsuge I., Tanaka A., Borres M.P., Urisu A. (2008). Utility of ovomucoid-specific IgE concentrations in predicting symptomatic egg allergy. J. Allergy Clin. Immunol..

[48] Rodríguez-Catalán J., González-Arias A.M., Casas A.V., Camacho G.D.R. (2021). Specific IgE levels as an outcome predictor in egg-allergic children. Allergol. Immunopathol..

[49] Quan P.L., Sabaté-Brescó M., D’Amelio C.M., Pascal M., García B.E., Gastaminza G., Blanca-López N., Alvarado M.I., Fernández J., Moya C. (2022). Validation of a commercial allergen microarray platform for specific immunoglobulin E detection of respiratory and plant food allergens. Ann. Allergy Asthma Immunol..

