# Supplementary material for: Oral Food Challenges to Milk and Egg in Children: Associations with Skin Prick Tests and IgE Sensitization Profiles
Source: Nutrients. 2026 Jul 3;18(13):2157. doi: 10.3390/nu18132157 (PMC13363956; doi:10.3390/nu18132157)
Supplement: Supplementary file 1 [file nutrients-18-02157-s001.zip › nutrients-4380747-supplementary.pdf]

Supplementary materials

Table S1. Comparison of total IgE levels and sIgE-to-total IgE ratios, both determined using the ALEX2 platform, in children with positive and negative OFC outcomes to baked milk

**Parameter**	**OFC +**	**OFC -**	**p-value**
tIgE ALEX2	1196.5 (204.8–4080.0)	918.0 (240.0–3383.0)	0.983
sIgE milk extract / tIgE ALEX2	9.81 (2.09–38.78) $\times 10^{-3}$	2.19 (1.31–9.25) $\times 10^{-3}$	0.096
sIgE α-lactalbumin / tIgE ALEX2	3.88 (1.30–5.36) $\times 10^{-3}$	1.61 (0.64–5.88) $\times 10^{-3}$	0.546
sIgE β-lactoglobulin / tIgE ALEX2	6.78 (1.03–22.25) $\times 10^{-3}$	0.72 (0.26–2.51) $\times 10^{-3}$	0.079
sIgE casein / tIgE ALEX2	6.57 (1.63–44.14) $\times 10^{-3}$	2.56 (0.95–8.71) $\times 10^{-3}$	0.233

Median (IQR); Mann-Whitney U test. Ratios multiplied by 10^{-3} for readability.

Table S2. Two-predictor logistic regression models for predicting positive OFC to baked milk

Model type	Model	AUC apparent	AUC corrected (boot)	Optimism	Max P(OFC+)	n >= 50%	n >= 75%	n >= 90%	n >= 95%
sIgE + sIgE	sIgE milk extract ImmunoCAP + sIgE casein ImmunoCAP	0.914 (0.815-1.000)	0.892	0.022	0.97	8	5	2	2
SPT + sIgE	SPT milk extract + sIgE casein ImmunoCAP	0.888 (0.772-1.000)	0.868	0.020	0.91	11	4	1	0
sIgE + sIgE	sIgE α-lactalbumin ImmunoCAP + sIgE casein ImmunoCAP	0.884 (0.769-0.999)	0.856	0.028	0.93	8	3	2	0
sIgE + sIgE	sIgE β-lactoglobulin ImmunoCAP + sIgE casein ImmunoCAP	0.884 (0.769-0.999)	0.856	0.028	0.96	7	2	2	2
sIgE + sIgE	sIgE milk extract ImmunoCAP + sIgE α-lactalbumin ImmunoCAP	0.862 (0.724-1.000)	0.832	0.030	0.87	5	2	0	0
sIgE + sIgE	sIgE milk extract ALEX2 + sIgE β-lactoglobulin ALEX2	0.852 (0.716-0.988)	0.820	0.032	0.87	7	3	0	0

AUC apparent: from the fitted model on original data (95% CI, DeLong). AUC corrected: bootstrap optimism-corrected AUC (1000 runs, Efron-Gong procedure). Optimism: difference between apparent and corrected AUC (> 0.05 suggests overfitting). Max P(OFC+): maximum predicted probability in the dataset. n >= X%: number of patients with predicted probability at or above threshold X. sIgE values log2-transformed in models. sIgE + sIgE models combine two components from the same platform only (ImmunoCAP or ALEX2, not mixed).

Table S3. Comparison of total IgE levels and sIgE-to-total IgE ratios, both determined using the ALEX2 platform, in children with positive and negative OFC outcomes to raw milk

Parameter	OFC +	OFC -	p-value
tIgE ALEX2	760.0 (276.0–1867.0)	384.0 (197.0–2531.0)	0.878
sIgE milk extract / tIgE ALEX2	1.55 (0.36–6.78) $\times 10^{-3}$	0.51 (0.26–1.03) $\times 10^{-3}$	0.219
sIgE α-lactalbumin / tIgE ALEX2	1.28 (0.19–5.88) $\times 10^{-3}$	0.51 (0.17–0.99) $\times 10^{-3}$	0.283
sIgE β-lactoglobulin / tIgE ALEX2	1.92 (0.60–3.56) $\times 10^{-3}$	0.42 (0.19–1.78) $\times 10^{-3}$	0.0776
sIgE casein / tIgE ALEX2	0.84 (0.13–7.32) $\times 10^{-3}$	0.38 (0.22–1.80) $\times 10^{-3}$	0.565

Median (IQR); Mann-Whitney U test. Ratios multiplied by 10^3 for readability

Table S4. Two-predictor logistic regression models for predicting positive OFC to raw milk

Model type	Model	AUC apparent	AUC corrected (boot)	Optimism	Max P(OFC+)	n >= 50%	n >= 75%	n >= 90%	n >= 95%
SPT + sIgE	SPT raw milk + sIgE α-lactalbumin ImmunoCAP	0.744 (0.569-0.920)	0.704	0.040	0.82	12	1	0	0
SPT + sIgE	SPT raw milk + sIgE β-lactoglobulin ImmunoCAP	0.740 (0.569-0.910)	0.697	0.043	0.83	13	2	0	0
SPT + sIgE	SPT raw milk + sIgE β-lactoglobulin ALEX2	0.741 (0.556-0.925)	0.701	0.040	0.87	11	3	0	0
sIgE + sIgE	sIgE milk extract ImmunoCAP + sIgE α-lactalbumin ImmunoCAP	0.735 (0.564-0.906)	0.698	0.037	0.77	14	3	0	0
sIgE + sIgE	sIgE milk extract ALEX2 + sIgE β-lactoglobulin ALEX2	0.765 (0.587-0.943)	0.723	0.042	0.88	12	2	0	0
sIgE + sIgE	sIgE α-lactalbumin ALEX2 + sIgE β-lactoglobulin ALEX2	0.767 (0.594-0.940)	0.725	0.042	0.91	11	2	2	0

AUC apparent: from the fitted model on original data (95% CI, DeLong). AUC corrected: bootstrap optimism-corrected AUC (1000 runs, Efron-Gong procedure). Optimism: difference between apparent and corrected AUC (> 0.05 suggests overfitting). Max P(OFC+): maximum predicted probability in the dataset. n >= X%: number of patients with predicted probability at or above threshold X. sIgE values log2-transformed in models. sIgE + sIgE models combine two components from the same platform only (ImmunoCAP or ALEX2, not mixed). The results of lysozyme-sIgE and serum albumin-sIgE were excluded from analysis due to >50% and >70% of values at limit of detection, respectively.

Table S5. Quantitative and qualitative agreement between ALEX2 and ImmunoCAP for results of milk components-sIgE (combined raw milk + baked milk cohorts, n=85)

Component	n	Spearman rho (95% CI)	p-value (Spearman)	Cohen kappa (95% CI)	Agreement n (%)	Both sensitized	Both non-sensitized	IC only	ALEX2 only
Milk extract	52	0.783 (0.616 to 0.887)	<0.001	0.484 (0.202 to 0.766)	44 (84.6%)	39	5	8	0
α-lactalbumin	52	0.899 (0.821 to 0.943)	<0.001	0.840 (0.691 to 0.990)	48 (92.3%)	29	19	3	1
β-lactoglobulin	52	0.874 (0.791 to 0.927)	<0.001	0.802 (0.637 to 0.967)	47 (90.4%)	28	19	2	3
casein	53	0.862 (0.764 to 0.923)	<0.001	0.385 (0.129 to 0.641)	41 (77.4%)	35	6	11	1

n: patients with both platforms available. Spearman rho with bootstrap 95% CI (2000 runs). Cohen's kappa based on platform-specific sensitization thresholds: 0.35 kUA/L for ImmunoCAP and 0.30 kUA/L for ALEX2 (manufacturer's limit of detection). Agreement: percentage of concordant classifications (both sensitized or both non-sensitized). Analysis performed on combined raw milk (n=43) and baked milk (n=42) cohorts; sensitization status is independent of the form of challenge. IC-ImmunoCAP

Table S6. Predictive performance comparison (paired): ALEX2 vs ImmunoCAP (baked milk cohort)

Component	n	AUC IC	AUC ALEX	delta AUC (ALEX - IC) 95% CI	DeLong p-value
Milk extract	24	0.805 (0.629-0.980)	0.887 (0.756-1.000)	+0.082 (-0.044 to +0.252)	0.238
α -lactalbumin	24	0.681 (0.424-0.937)	0.672 (0.414-0.931)	-0.008 (-0.090 to +0.070)	0.799
β -lactoglobulin	24	0.765 (0.557-0.973)	0.828 (0.590-1.000)	+0.063 (-0.111 to +0.261)	0.484
casein	25	0.853 (0.701-1.000)	0.912 (0.797-1.000)	+0.059 (-0.037 to +0.198)	0.297

AUC with 95% CI (DeLong). delta AUC: difference ALEX - IC with bootstrap 95% CI. DeLong paired test for comparing AUCs of correlated ROC curves. CI including 0 indicates no evidence of difference between platforms.

Table S7. Predictive performance comparison (paired): ALEX2 vs ImmunoCAP (raw milk cohort)

Component	n	AUC IC	AUC ALEX	delta AUC (ALEX - IC) 95% CI	DeLong p-value
Milk extract	28	0.722 (0.518-0.926)	0.668 (0.462-0.875)	-0.053 (-0.260 to +0.133)	0.583
α -lactalbumin	28	0.701 (0.460-0.941)	0.717 (0.506-0.928)	+0.016 (-0.117 to +0.150)	0.803
β -lactoglobulin	28	0.610 (0.373-0.846)	0.701 (0.495-0.906)	+0.091 (-0.048 to +0.231)	0.179
casein	28	0.631 (0.406-0.856)	0.647 (0.424-0.870)	+0.016 (-0.184 to +0.193)	0.867

AUC with 95% CI (DeLong). delta AUC: difference ALEX - IC with bootstrap 95% CI. DeLong paired test for comparing AUCs of correlated ROC curves. CI including 0 indicates no evidence of difference between platforms.

Table S8. Comparison of total IgE levels and sIgE-to-total IgE ratios, both determined using the ALEX2 platform, in children with positive and negative OFC outcomes to boiled egg

Parameter	OFC +	OFC -	p-value
tIgE ALEX2	1143.0 (226.2–2844.5)	304.0 (128.0–1609.0)	0.240
sIgE egg white extract / tIgE ALEX2	4.11 (1.63–8.80) $\times 10^{-3}$	1.75 (0.99–3.87) $\times 10^{-3}$	0.134
sIgE egg yolk extract / tIgE ALEX2	0.39 (0.17–1.36) $\times 10^{-3}$	0.56 (0.15–1.09) $\times 10^{-3}$	0.915
sIgE ovomucoid/ tIgE ALEX2	1.95 (0.54–5.35) $\times 10^{-3}$	1.03 (0.47–2.33) $\times 10^{-3}$	0.229
sIgE ovalbumin / tIgE ALEX2	3.05 (0.50–5.68) $\times 10^{-3}$	1.35 (0.52–3.28) $\times 10^{-3}$	0.454
sIgE ovotransferrin / tIgE ALEX2	0.80 (0.29–1.98) $\times 10^{-3}$	0.51 (0.26–1.09) $\times 10^{-3}$	0.504

Median (IQR); Mann-Whitney U test. Ratios multiplied by 10^{-3} for readability.

Table S9. Two-predictor logistic regression models for predicting positive OFC to boiled egg

Model type	Model	AUC apparent	AUC corrected (boot)	Optimism	Max P(OFC+)	n >= 50%	n >= 75%	n >= 90%	n >= 95%
SPT + sIgE	SPT egg white extract + sIgE egg white extract ALEX2	0.797 (0.644-0.951)	0.778	0.019	0.83	15	2	0	0
SPT + sIgE	SPT egg yolk extract + sIgE egg yolk extract ALEX2	0.800 (0.661-0.939)	0.769	0.031	0.97	12	5	2	1
SPT + sIgE	SPT egg white raw + sIgE egg white extract ALEX2	0.791 (0.642-0.939)	0.762	0.029	0.88	14	2	0	0
SPT + sIgE	SPT egg yolk extract + sIgE egg white extract ALEX2	0.787 (0.636-0.938)	0.754	0.033	0.90	16	1	0	0
SPT + sIgE	SPT egg white raw + sIgE ovomucoid ALEX2	0.768 (0.611-0.925)	0.740	0.028	0.89	12	3	0	0
SPT + sIgE	SPT egg white raw + sIgE ovotransferrin ALEX2	0.773 (0.618-0.928)	0.739	0.034	0.92	11	3	1	0
SPT + sIgE	SPT egg white raw + sIgE ovalbumin ALEX2	0.768 (0.613-0.923)	0.734	0.034	0.87	12	3	0	0
SPT + sIgE	SPT egg yolk raw + sIgE egg yolk extract ALEX2	0.774 (0.619-0.928)	0.732	0.042	0.84	9	4	0	0

AUC apparent: from the fitted model on original data (95% CI, DeLong). AUC corrected: bootstrap optimism-corrected AUC (1000 runs, Efron-Gong procedure). Optimism: difference between apparent and corrected AUC (> 0.05 suggests overfitting). Max P(OFC+): maximum predicted probability in the dataset. n >= X%: number of patients with predicted probability at or above threshold X. sIgE values log2-transformed in models. sIgE + sIgE models combine two components from the same platform only (ImmunoCAP or ALEX, not mixed).

Table S10. Comparison of total IgE levels and sIgE-to-total IgE ratios, both determined using the ALEX2 platform, in children with positive and negative OFC outcomes to baked egg

Parameter	OFC +	OFC -	p-value
tIgE ALEX2	1916.0 (839.0–2129.0)	867.0 (238.0–2181.0)	0.419
sIgE egg white extract / tIgE ALEX2	11.25 (4.94–34.26) $\times 10^{-3}$	5.52 (1.78–16.39) $\times 10^{-3}$	0.192
sIgE egg yolk extract / tIgE ALEX2	0.66 (0.34–1.40) $\times 10^{-3}$	0.54 (0.18–1.18) $\times 10^{-3}$	0.416
sIgE ovomucoid / tIgE ALEX2	3.11 (2.46–14.00) $\times 10^{-3}$	1.87 (0.99–9.48) $\times 10^{-3}$	0.304
sIgE ovalbumin / tIgE ALEX2	10.78 (4.40–25.15) $\times 10^{-3}$	3.52 (1.44–11.39) $\times 10^{-3}$	0.069
sIgE ovotransferrin / tIgE ALEX2	2.79 (0.64–3.11) $\times 10^{-3}$	0.71 (0.24–2.24) $\times 10^{-3}$	0.174
sIgE lysozyme / tIgE ALEX2	3.91 (2.73–6.42) $\times 10^{-3}$	0.92 (0.18–3.88) $\times 10^{-3}$	0.186
sIgE serum albumin / tIgE ALEX2	0.12 (0.05–0.24) $\times 10^{-3}$	0.14 (0.05–0.67) $\times 10^{-3}$	0.578

Median (IQR); Mann-Whitney U test. Ratios multiplied by 10^{-3} for readability.

Table S11. Two-predictor logistic regression models for predicting positive OFC to baked egg

Model type	Model	AUC apparent	AUC corrected (boot)	Optimism	Max P(OFC+)	n >= 50%	n >= 75%	n >= 90%	n >= 95%
sIgE + sIgE	sIgE ovomucoid ALEX2 + sIgE ovalbumin ALEX2	0.875 (0.765-0.985)	0.848	0.027	0.64	6	0	0	0
sIgE + sIgE	sIgE ovalbumin ALEX2+ sIgE ovotransferrin ALEX2	0.857 (0.709-1.000)	0.823	0.034	0.65	4	0	0	0
SPT + sIgE	SPT egg yolk raw + sIgE ovalbumin ALEX2	0.859 (0.716-1.000)	0.821	0.038	0.62	2	0	0	0
sIgE + sIgE	sIgE egg white extract ALEX2 + sIgE ovalbumin ALEX2	0.853 (0.722-0.983)	0.817	0.036	0.66	5	0	0	0
sIgE + sIgE	sIgE egg yolk extract ALEX2 + sIgE ovalbumin ALEX2	0.843 (0.703-0.983)	0.810	0.034	0.62	5	0	0	0
SPT + sIgE	SPT egg white extract + sIgE egg white extract ALEX2	0.862 (0.730-0.994)	0.809	0.052	0.89	5	1	0	0
SPT + sIgE	SPT egg yolk raw + sIgE ovotransferrin ALEX2	0.852 (0.638-1.000)	0.809	0.043	0.61	2	0	0	0
sIgE + sIgE	sIgE egg white extract ALEX + sIgE ovomucoid ALEX2	0.857 (0.722-0.993)	0.806	0.051	0.58	5	0	0	0
SPT + sIgE	SPT egg white extract + sIgE ovalbumin ALEX2	0.844 (0.712-0.976)	0.802	0.042	0.86	3	1	0	0
SPT + sIgE	SPT egg white extract + sIgE egg yolk ImmunoCAP	0.818 (0.650-0.986)	0.800	0.018	0.87	8	2	0	0

AUC apparent: from the fitted model on original data (95% CI, DeLong). AUC corrected: bootstrap optimism-corrected AUC (1000 runs, Efron-Gong procedure). Optimism: difference between apparent and corrected AUC (>

0.05 suggests overfitting). Max P(OFC+): maximum predicted probability in the dataset. n >= X%: number of patients with predicted probability at or above threshold X. sIgE values log2-transformed in models. sIgE + sIgE models combine two components from the same platform only (ImmunoCAP or ALEX2, not mixed).

Table S12. Quantitative and qualitative agreement between results of ALEX2 and ImmunoCAP for results of egg extracts-sIgE (combined boiled egg + baked egg cohorts, n=108)

Component	n	Spearman rho (95% CI)	p-value (Spearman)	Cohen kappa (95% CI)	Agreement n (%)	Both sensitized	Both non-sensitized	IC only	ALEX2 only
Egg white extract	62	0.874 (0.784 to 0.927)	<0.001	0.484 (0.183 to 0.786)	54 (87.1%)	49	5	6	2
Egg yolk extract	62	0.853 (0.752 to 0.911)	<0.001	0.445 (0.266 to 0.625)	44 (71.0%)	28	16	18	0

n: patients with both platforms available. Spearman rho with bootstrap 95% CI (2000 runs). Cohen's kappa based on platform-specific sensitization thresholds: 0.35 kUA/L for ImmunoCAP and 0.30 kUA/L for ALEX2 (manufacturer's limit of detection). Agreement: percentage of concordant classifications (both sensitized or both non-sensitized). Analysis performed on combined boiled egg (n=54) and baked egg (n=54) cohorts; sensitization status is independent of the form of challenge. Note: molecular allergen components (ovomucoid, ovalbumin, ovotransferrin) were measured exclusively on ALEX2 and are not included in this comparison.

Table S13. Predictive performance comparison (paired): ALEX2 vs ImmunoCAP for egg extracts (boiled egg cohort)

Component	n	AUC IC	AUC ALEX	delta AUC (ALEX - IC) 95% CI	DeLong p-value
Egg white extract	32	0.652 (0.445-0.859)	0.690 (0.496-0.883)	+0.037 (-0.059 to +0.146)	0.42
Egg yolk extract	32	0.667 (0.451-0.883)	0.694 (0.505-0.883)	+0.027 (-0.097 to +0.163)	0.667

AUC with 95% CI (DeLong). delta AUC: difference ALEX - ImmunoCAP with bootstrap 95% CI. DeLong paired test for comparing AUCs of correlated ROC curves. CI including 0 indicates no evidence of difference between platforms.

Table S14. Predictive performance comparison (paired): ALEX2 vs ImmunoCAP for egg extracts (baked egg cohort)

Component	n	AUC IC	AUC ALEX	delta AUC (ALEX - IC) 95% CI	DeLong p-value
Egg white extract	30	0.616 (0.359-0.873)	0.688 (0.406-0.970)	+0.072 (-0.056 to +0.230)	0.264
Egg yolk extract	30	0.776 (0.568-0.984)	0.840 (0.639-1.000)	+0.064 (-0.248 to +0.355)	0.65

AUC with 95% CI (DeLong). delta AUC: difference ALEX - ImmunoCAP with bootstrap 95% CI. DeLong paired test for comparing AUCs of correlated ROC curves. CI including 0 indicates no evidence of difference between platforms.
